# Interactions Between Carbon Metabolism and Photosynthetic Electron Transport in a *Chlamydomonas reinhardtii* Mutant Without CO_2_ Fixation by RuBisCO

**DOI:** 10.3389/fpls.2022.876439

**Published:** 2022-04-28

**Authors:** Maureen Saint-Sorny, Pawel Brzezowski, Stéphanie Arrivault, Jean Alric, Xenie Johnson

**Affiliations:** ^1^CEA, CNRS, UMR 7265, BIAM, CEA Cadarache, Aix-Marseille Université, Saint-Paul-lez-Durance, France; ^2^Max Planck Institute of Molecular Plant Physiology, Potsdam, Germany

**Keywords:** RuBisCO, chlorophyll fluorescence, acetate assimilation, Calvin Benson Bassham cycle, TCA cycle, photosynthetic yield of PSII, photosynthetic control, reducing power

## Abstract

A *Chlamydomonas reinhardtii* RuBisCO-less mutant, Δ*rbcL*, was used to study carbohydrate metabolism without fixation of atmospheric carbon. The regulatory mechanism(s) that control linear electron flow, known as “photosynthetic control,” are amplified in Δ*rbcL* at the onset of illumination. With the aim to understand the metabolites that control this regulatory response, we have correlated the kinetics of primary carbon metabolites to chlorophyll fluorescence induction curves. We identify that Δ*rbcL* in the absence of acetate generates adenosine triphosphate (ATP) *via* photosynthetic electron transfer reactions. Also, metabolites of the Calvin Benson Bassham (CBB) cycle are responsive to the light. Indeed, ribulose 1,5-bisphosphate (RuBP), the last intermediate before carboxylation by Ribulose-1,5-bisphosphate carboxylase-oxygenase, accumulates significantly with time, and CBB cycle intermediates for RuBP regeneration, dihydroxyacetone phosphate (DHAP), pentose phosphates and ribose-5-phosphate (R5P) are rapidly accumulated in the first seconds of illumination, then consumed, showing that although the CBB is blocked, these enzymes are still transiently active. In opposition, in the presence of acetate, consumption of CBB cycle intermediates is strongly diminished, suggesting that the link between light and primary carbon metabolism is almost lost. Phosphorylated hexoses and starch accumulate significantly. We show that acetate uptake results in heterotrophic metabolism dominating phototrophic metabolism, with glyoxylate and tricarboxylic acid (TCA) cycle intermediates being the most highly represented metabolites, specifically succinate and malate. These findings allow us to hypothesize which metabolites and metabolic pathways are relevant to the upregulation of processes like cyclic electron flow that are implicated in photosynthetic control mechanisms.

## Introduction

Plants, algae, and cyanobacteria use light energy to synthesize organic compounds from CO_2_. This light driven electron transfer draws electrons from the photolysis of water, releasing oxygen as a byproduct. Unlike plants, the unicellular green alga *Chlamydomonas reinhardtii* has a flexible metabolism and can grow autotrophically using light and CO_2_ *via* photosynthesis, heterotrophically utilizing a chemical carbon source (acetate) or mixotrophically using both CO_2_ and acetate. This offers advantages to study mutants impaired in photosynthesis as cells retain chlorophyll along with the photosynthetic apparatus, and have rapid heterotrophic growth in darkness (Sasso et al., 2018). *C. reinhardtii* metabolism can optimize the energetic balance between the adenosine triphosphate (ATP) requirements for acetate uptake, oxidative phosphorylation, and photosynthesis, with the metabolic demands of the tricarboxylic acid (TCA) cycle and the Calvin Benson Bassham (CBB) cycle. Such metabolic flexibility results in strong interactions between photosynthesis and respiration with a number of studies striving to understand what controls the partitioning of ATP and nicotinamide adenine dinucleotide phosphate hydrogen (NADPH) between the two compartments (reviewed in Johnson and Alric, 2013).

The photosynthetic electron transport chain consists of two different electron flows that work simultaneously: the linear electron flow (LEF), which produces reducing power and ATP, and the cyclic electron flow (CEF), which only produces ATP. In order to assimilate carbon, the CBB cycle requires ATP and NADPH in a ratio of 3:2 that is not met by linear electron flow alone (Allen et al., 2011). Thus, regulatory mechanisms are required between the linear and cyclic flows to adjust the ATP/NADPH ratio. Under mixotrophic conditions, CEF activity and acetate uptake are tightly linked. Acetate uptake requires active ATP production and has the same action spectrum as PSI making a link to increased PSI activity and thus CEF (Wiessner, 1965; Gibbs et al., 1986). Furthermore, *C. reinhardtii* grown mixotrophically down regulates photosynthesis but still shows a high growth rate, due to the combination of acetate uptake and residual CO_2_ assimilation (Heifetz et al., 2000).

The redox state of the stroma as well as the electron transfer chain complexes and carriers are important determinants for rates of CEF. A comparative study of a wild-type with mutants inactivated for the CBB cycle provided evidence that the rate of P_700_ kinetics and thus CEF is controlled by the redox poise of the stroma (Alric et al., 2010). Furthermore, mutants without starch metabolism (*sta6*) were found to induce changes in the redox poise of the plastoquinone pool resulting in a twofold increase in the rate of CEF (Johnson and Alric, 2012). *sta6* showed a decreased photosynthetic activity compared to the wild-type under mixotrophic conditions with a slow rise in chlorophyll fluorescence attributed to a reduced plastoquinone pool and a slowing down of NADPH re-oxidation (Krishnan et al., 2015). In Takahashi et al. (2013), CEF as well as formation of supercomplexes were shown to be tuned by reducing conditions, independently from the migration of antenna proteins toward PSI or PSII (state transition). Strand et al. (2016) demonstrated a link between strongly reduced redox poise and activation of CEF specific to PGRL1/PGR5 in *Arabidopsis thaliana*. These studies showed that CEF correlated with changes or limitations to metabolism.

Ribulose-1,5-bisphosphate carboxylase-oxygenase (RuBisCO) is the key enzyme in the CBB cycle responsible for CO_2_ fixation. RuBisCO consists of two subunits, the large chain, encoded by the *rbcL* gene in the chloroplast, and the small chain, encoded by several genes in the nucleus. The Δ*rbcL* mutant lacks the large chain subunit and does not assemble functional RuBisCO enzyme (Johnson et al., 2010). Although RuBisCO-less mutants are devoid of CO_2_ fixation and photorespiration, the photosynthetic electron transport chain remains functional and Δ*rbcL* relies on its interactions between mitochondria and chloroplast to metabolize carbon present in other form like acetate. The absence of a functional CBB cycle in Δ*rbcL* results in accumulation of electrons along the photosynthetic chain. To maintain photosynthetic electron transfer in Δ*rbcL*, these electrons are transferred to the alternative electron acceptors and take the place of CO_2_ fixation. The alternative pathways include oxygen photoreduction catalyzed by the Mehler reaction, involve flavodiiron proteins in the chloroplast, oxidative phosphorylation, and alternative oxidases (Johnson et al., 2014). When washed of the acetate required for growth, a light inhibition of TCA cycle activity can be observed due to “competition” for cytosolic ADP between chloroplastic and oxidative phosphorylations (Gans and Rebeille, 1988).

During a light induction, the limitation at the acceptor side of PSI in Δ*rbcL* results in a slow rise in steady state chlorophyll fluorescence due to decreasing PSII activity (Johnson et al., 2010; Johnson, 2011). This rise is abolished in a double mutant Δ*rbcL pgr5*, indicating that PGR5 controls the fluorescence rise observed in Δ*rbcL*. Proton Gradient Regulation 5 (PGR5) has been shown to be involved in CEF and luminal acidification processes resulting in a dual activity for PGR5 described as “photosynthetic control” (Yamamoto and Shikanai, 2019). This consists of a downregulation of the electron flow between PSII and PSI by the slowing effect of proton gradient on cytochrome *b_6_f* electron transfer, and so contributes to photoprotection (Suorsa et al., 2012; Chaux et al., 2015, 2017; Dumas et al., 2016). Further studies on *pgr5* and its functional partner *pgrl1* showed that these mutants have increased and sustained mitochondrial-chloroplast interactions in both algae and plants (Torzillo et al., 2009; Tolleter et al., 2011; Scoma et al., 2012; Volgusheva et al., 2013; Dang et al., 2014; Steinbeck et al., 2015).

In this work, our aim was to identify metabolites that impact redox-activated photosynthetic regulatory mechanisms, ADP:ATP ratios and ADP partitioning between the chloroplast and other compartments. We do this by using a mutant without CO_2_ fixation because it has highly active alternative electron flow pathways, specifically related to PGR5 function, that combine to down-regulate linear electron flow in Δ*rbcL.* Here, we have compared Δ*rbcL* cells in the absence and presence of acetate, at the onset of illumination, when these regulatory mechanisms are active and easily measured. We have then correlated the kinetics of primary carbon metabolites to chlorophyll fluorescence induction curves to make a link between metabolites and metabolic pathways that stimulate down regulation of linear electron flow and those that maintain it.

## Materials and Methods

### Mutant Δ*rbcL* and Cell Culture

Strains derived from CC-137 were generated to make RuBisCO-less mutant, Δ*rbcL* (Johnson et al., 2010). The wild-type strain used in experiments is Jex 4, also derived from CC-137 (Johnson et al., 2014). *C. reinhardtii* strains were maintained on agar-solidified tris-acetate-phosphate (TAP) at low light intensities. Strains were grown in TAP-liquid cultures at 10 μmol photons m^–2^ s^–1^ in an incubator with shaking at 150 rotations per minute. Strains were grown in batch cultures, diluted three times and confirmed in exponential phase before resuspension at equal chlorophyll concentration, into fresh medium with or without acetate (washed once before resuspension), or with acetate but in absence of nitrogen (washed before resuspension).

### Growth Test on Solid Medium (Spot Test)

Growth tests were conducted by placing 25 μL of liquid cultures at a density of 1 × 10^6^ cells/mL onto solid medium and transferred to dark, or 10, 20, 40, 60, or 80 μmol photons m^–2^ s^–1^ illumination onto solid medium during a few days.

### Chlorophyll Fluorescence Analysis

Fluorescence and absorbance kinetics were performed using the JTS-10 spectrophotometer (BioLogic, Seyssinet-Pariset, France) as previously described (Joliot and Joliot, 2002). For fluorescence measurements, quantum yield of PSII is calculated as ϕ_*PSII*_ = (F_*M*_’ – F’)/F_*M*_’.

### Electrochromic Shift Measurements

Absorbance changes at 520 nm (ΔA_520*nm*_) were monitored using a JTS-10 spectrophotometer (BioLogic, Seyssinet-Pariset, France), according to the method detailed in Mathiot and Alric (2021). At steady-state, the building of the electrochemical proton gradient by photochemistry is equal to the destruction of the proton gradient for ATP synthesis. The post-illumination decay of ΔA_520*nm*_ is therefore a proxy of the steady state electron transport flow rate through PSI and PSII, ETR_*PSI+PSII*_. ETR_*PSI+PSII*_ was normalized on the amplitude of a single turnover saturating flash (1 charge separated across the membrane per photosystem).

### Chlorophyll and Starch Quantification

Chlorophyll content in Δ*rbcL* and WT was estimated using modified method from Porra et al. (1989). Briefly, 5 × 10^6^ cells were collected, centrifuged (16,000 × *g*) for 10 min, resuspended in methanol (1 mL) for chlorophyll extraction, and placed at –20°c for 20 min for chlorophyll extraction. Chlorophyll concentration was estimated directly on supernatants by measuring absorbance at 665, 652, and 750 nm.

Following the chlorophyll measurements, the remaining cell pellet was used to determine starch content using a method modified from Klein and Betz (1978). The pellets were dried and resuspended in 400 μL H_2_O. Samples were autoclaved at 120°C for 20 min and cooled. Starch was then digested to glucose with 200 μL of amyloglucosidase (1 U/mL) solution (Starch Assay Reagent, Sigma–Aldrich, Saint-Louis, MO, United States) in 60°C for 1.5 h and cooled. Samples were centrifuged at 16,000 × g for 3 min and glucose content was determined in the supernatant using an automated sugar analyzer (YSI model 2700 Select, Yellow springs, OH, United States).

### Experimental Set Up for Metabolite Analysis

Cultured cells in exponential phase were resuspended at the same concentration, 1 × 10^7^ cells/mL and cultures were verified for reproducible differences in chlorophyll fluorescence kinetics using Joliot type spectrometer (JTS) for electron transfer kinetics. Cells were counted using a Cell counter (Beckmann Coulter, Brea, CA, United States), chlorophyll was quantified and 60 mL of culture were transferred to a “lollipop” set-up. Cells were incubated in total darkness for 1 min and sampled before the light treatment. Cells were illuminated with 300 μmol photons m^–2^ s^–1^ using Xenon lamps on both sides of the culture and directed onto the quenching tubes.

### Harvesting Procedure

A quenching solution consisting of 70% methanol was cooled down to –60° to –70°C cold ethanol, which was maintained cool by dry ice (Bölling and Fiehn, 2005; Kempa et al., 2009) in a dewar container. The continuously illuminated culture was injected into the quenching solution by a syringe (5 mL; 1:2 = culture: quenching solution) at various time points. Temperature was checked regularly to assure that temperature was kept below –20°C during the whole procedure. The material was transferred to –80°C freezer before metabolite extraction.

### Sample Extraction and Metabolite Measurements

Metabolite profiling was conducted using anion-exchange high performance liquid chromatography coupled to tandem mass spectrometry (LC-MS/MS). Water-soluble metabolites were extracted using a slightly modified protocol as previously published (Lunn et al., 2006; Arrivault et al., 2009) which was previously reported in Mettler et al. (2014). Quenched algal material (525 μL) was added to 105 μL precooled chloroform and was vigorously vortexed in the presence of glass beads (final ratio C:W:M = 1:5.3:2.3). The cells were exposed to three cycles of thaw-freeze to break cells completely. The aqueous fraction was collected after centrifugation (5 min, 13,500 rpm, 4°C), and the chloroform fraction was washed two additional times with 560 μL ice-cold water. The three aqueous fractions were combined and freeze-dried overnight (Alpha 2-4; Christ). Samples were resuspended in water and filtered with Ultracel-10 multiscreen filter plates (Millipore, Burlington, MA, United States). An equivalent of 70–80 μL of culture was analyzed per single LC-MS/MS run. Stable isotopically labeled internal standards were added to correct for matrix effects (Arrivault et al., 2015). The enzymatic assay used for 3-PGA detection was performed according to Mettler et al. (2014).

### Statistical Analysis

Statistical analyses were performed using Rstudio software. Significant differences in metabolite quantities were determined using either Kruskal–Wallis, Dunn test, Wilcoxon–Mann–Whitney test or one way ANOVA. Comparisons of groups over time were carried out with either two-way ANOVA, Dunn test or pairwise Wilcoxon (everytime with Bonferroni posttest and adjustments). For the heatmaps, data has been normalized by metabolite and heatmaps have been generated by the heatmaply package on Rstudio. Color is based on correlation coefficient according to the color scale indicated.

## Results

### Δ*rbcL* Is a Non-phototrophic and Light-Sensitive Mutant Accumulating High Levels of Starch

**FIGURE 1:**
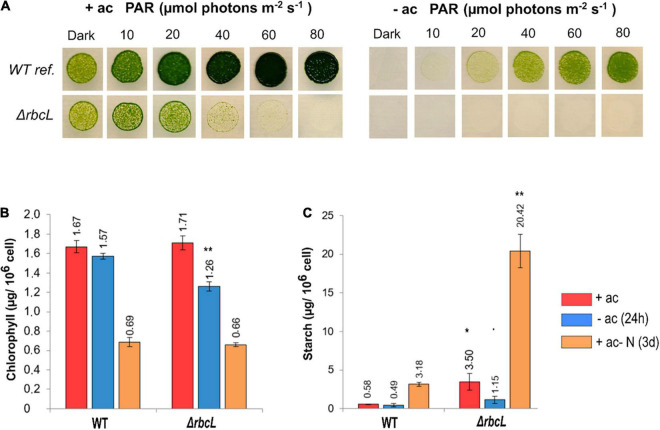
Characteristics of the RuBisCO knockout strain Δ*rbcL.* **(A)** Spot test to compare growth and photosensitivity of WT and Δ*rbcL* on agar plates with (+ac) or without (–ac) acetate at six increasing light intensities. **(B)** Total chlorophyll content and **(C)** Starch accumulation under different conditions for WT and Δ*rbcL* in low light (10 μmol photons m^–2^ s^–1^), grown with acetate, without acetate during 24 h, or with acetate but in absence of nitrogen during 3 days. Biological replicates (*n* = 3) with standard error. Pairwise *t*-test were performed between WT and Δ*rbcL* (one dot, *p* < 0.1; one asterisk, *p* < 0.05; two asterisks, *p* < 0.01).

The growth characteristics of Δ*rbcL* were compared to the wild-type reference strain. The Δ*rbcL* strain is light sensitive when grown on acetate at light intensities stronger than 20 μmol photons m^–2^ s^–1^. Δ*rbcL* cannot grow without acetate because CO_2_ fixation does not occur without RuBisCO (Figure 1A). It can however be maintained for days when resuspended in a minimal medium without acetate if cultivated under low light (10–20 μmol photons m^–2^ s^–1^) and with sufficient oxygenation (Johnson et al., 2010).

Accumulation of total chlorophyll and starch were determined under different conditions. Cells were grown with acetate, and then resuspended at the same cell density without acetate for 1 day, or in acetate medium without a nitrogen source for 3 days. Growing cells without nitrogen stops the cell cycle and thus cell division and cells are reprogrammed to stock a maximal quantity of starch reserves. In the presence of acetate, the chlorophyll content is similar between Δ*rbcL* and the wild-type (Figure 1B). The Δ*rbcL* mutant grown without acetate for 24 h shows a 20% reduction in the chlorophyll content compared to the wild-type (Figure 1B). After 3 days of growth without nitrogen, both strains show a 60% reduction in chlorophyll content, normal under these conditions (Figure 1B). Starch accumulation is six times greater in Δ*rbcL* than in the wild-type, both with acetate and when nitrogen is removed from the medium (Figure 1C). The maximal quantity of starch is interesting because it shows that Δ*rbcL* capacity to accumulate high levels of starch is constitutively rewired and not condition dependent. In acetate-free medium, Δ*rbcL* rapidly mobilizes the starch reserves as deduced by a threefold reduction in starch over the 24 h period (Figure 1C).

### Δ*rbcL* Electron Transport Is Affected by the Presence of Acetate, Linking Chlorophyll Fluorescence Kinetics to Changes in Carbon Metabolism

Over a 5-min period of illumination (300 μmol photons m^–2^ s^–1^), the chlorophyll fluorescence kinetics of wild-type cells grown in acetate show a constant photosynthetic activity (F_*M*′_-F′). The wild-type grown with or without acetate behaves similarly, with a slight quenching of F_*M*_ after the illumination in the absence of acetate.

**FIGURE 2:**
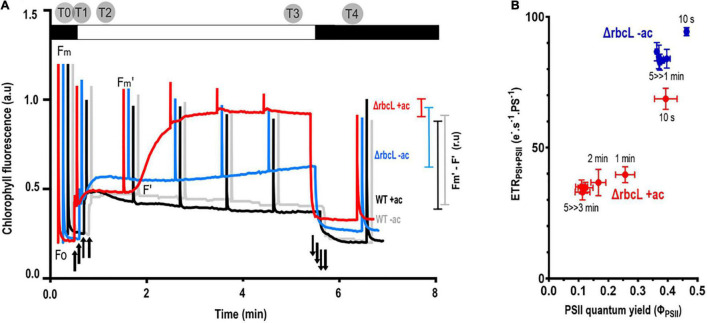
Acetate has significant effects on photosynthetic activity in Δ*rbcL*. Photosynthetic yield as a proxy for photosynthetic electron transport. **(A)** Typical chlorophyll fluorescence kinetics for wild-type cells cultured with acetate (+ ac) in black, without acetate (–ac) in gray, and comparison with Δ*rbcL* chlorophyll fluorescence kinetics when grown with acetate (+ac, in red) or resuspended in medium without acetate (–ac, in blue). Cells were dark adapted and subjected to 300 μmol photons m^–2^ s^–1^ actinic light over 5 min with saturating flashes to probe photosynthetic yield in the light shown as colored bars on the right of the figure (F_*m*′_-F′). Light switched on and off is denoted respectively by up and down arrows. Time points for the metabolite sampling are represented on the upper part of the graph. **(B)** ETR_*PSI+PSII*_ as a function of φ_*PSII*_ over a 5 min illumination in Δ*rbcL* in acetate-replete (dark, red symbols) or acetate-deplete (open, blue symbols) medium. The time in the light before taking a measurement is shown by the labels. φ_*PSII*_ values were derived from the chlorophyll fluorescence kinetics as in Figure 2A. ETR_*PSI* + *PSII*_ was measured as the decay rate of absorbance ΔA_520*nm*_ (electrochromic shift of carotenoids, ECS).

In contrast, Δ*rbcL* grown in acetate shows a rise in fluorescence (F’) during the illumination, toward the maximum fluorescence (F_*M*’_), which indicated that the photosynthetic activity is significantly decreased (Figure 2A). In the absence of acetate, Δ*rbcL* maintains a photosynthetic activity that is significantly higher than in the presence of acetate (Figure 2A). Figure 2B shows a sampling at different time points of the fluorescence parameter Φ_*PSII*_ (F_*M*’_-F’)/F_*M*’_ relative to the total electron flow rate through both PSII and PSI (ETR_*PSII* + *PSI*_). The latter is formally identical to ν_*H+*_ (efflux through the membrane) (Avenson et al., 2005), but normalized to photosystem turnover (see section “Materials and Methods”). In the absence of acetate, consistent with a large PSII quantum yield, the electron transport rate remains high. In the presence of acetate, Φ_*PSII*_ and ETR_*PSII* + *PSI*_ decrease but not to the same extent. Total electron flow is maintained even when Φ_*PSII*_ is very low, suggesting a larger contribution from PSI and lending support to significant CEF activity in Δ*rbcL* in the presence of acetate.

To identify carbohydrates that regulate and influence the decrease in photosynthetic yield in Δ*rbcL* grown in acetate, samples for metabolite profiling were taken at different time points over a transient light induction. The Δ*rbcL* cells were grown in acetate containing medium, and then resuspended in medium with or without acetate and incubated in low light for 2–3 days. The chlorophyll fluorescence kinetics of the cultures were monitored and after 2 or 3 days, the cells were transferred to a flask bubbled with air that guaranteed a homogenous illumination. Cultures were incubated 5 min in the dark and then exposed to high light for 5 min. Samples were rapidly quenched at different time points (Dark, 10 s, 1 min, 5 min, 1 min after return in the dark as represented in Figure 2) into freezing cold methanol. The time points were chosen since they represent different key moments in the transient and steady state response of photosynthesis to a light treatment. Two dark treatments were chosen to make reference to the metabolic state before and after light. The quenched samples were treated and analyzed by LC-MS/MS (cf. “Materials and Methods”). A list of the 23 chosen metabolites (Supplementary Table 1) has representative components of the CBB, TCA and glyoxylate cycles, as well as metabolites required for starch production (gluconeogenesis), key amino acids, and energy carrying molecules.

**FIGURE 3:**
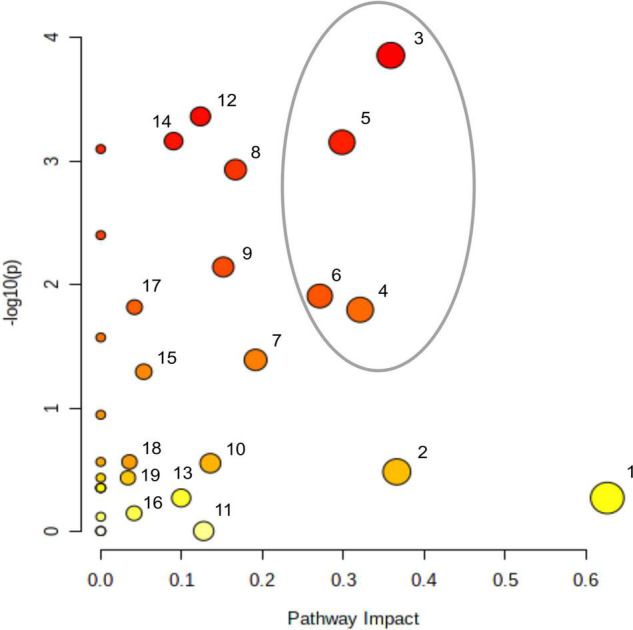
Impact of acetate on metabolites accumulations and on cell metabolism. There are four replicates per metabolite per medium, samples have been taken after a 5 min light treatment (300 μmol photons m^–2^ s^–1^). Metabolites were quantified per LC-MS/MS after quenching and extraction. Impact of accumulating metabolites on cell metabolism analyzed using MetaboAnalyst 5.0 (https://www.metaboanalyst.ca), sorted by the *P*-value; **(1)** alanine, aspartate, and glutamate metabolism, **(2)** pentose phosphate metabolism, **(3)** amino sugar and nucleotide sugar metabolism, **(4)** TCA cycle, **(5)** starch and sucrose metabolism, **(6)** glyoxylate and dicarboxylate metabolism, **(7)** carbon fixation, **(8)** pentoses and gluconarate interconversions, **(9)** nicotinate and nicotinamide metabolism, **(10)** purine metabolism, **(11)** arginine and proline metabolism, **(12)** galactose metabolism, **(13)** arginine biosynthesis, **(14)** glycerolipid metabolism, **(15)** glutathione metabolism, **(16)** glycine, serine, and threonine metabolism, **(17)** pyruvate metabolism, **(18)** sulfur metabolism, **(19)** fructose and mannose metabolism. The gray ellipse represents the most significantly affected pathways.

In order to compare the effect of acetate on the central carbon metabolism in Δ*rbcL*, different statistical analyses were conducted on the overall results (Kruskal, Dunn, Wilcox, Anova: refer to Supplementary Table 2 for the explanations of each statistical test), using the null hypothesis that there were no differences for all 23 metabolites between the different culture conditions. Overall, the 23 key metabolites are significantly affected by the presence of acetate as the calculated *p*-values are all less than 0.05 (in bold in Table 1 and Supplementary Table 3). We showed the high significance of the influence of acetate on the metabolites from diverse metabolic pathways. In Figure 3, Metaboanalyst^1^ was used to assess the impact of given metabolites on a particular pathway. Using this analysis, the matched pathways are shown as the common logarithm of *p*-values on the *y*-axis (yellow to red shows increasing significance) and pathway impact values (from topology analysis) on the *x*-axis (the impact increases with the dot’s size). Pathways with most significant differences in metabolite levels between culture conditions are found in the top and to the right in this graph (large red dots encircled by a gray ellipse): amino sugar and nucleotide sugar metabolism (3), TCA cycle (4), starch metabolism (5), and glyoxylate metabolism (6).

**TABLE 1:** Statistical analysis of the comparison of Δ*rbcL* growth in the presence and absence of acetate and its influence on central C metabolism. Different statistical analysis (Kruskal, Dunn, Wilcox, aov) show that 23 key metabolites are significantly affected by the presence of acetate.

Statistical test	*p*-value	*p*-significance
Kruskal–Wallis	5,24e-10	****
Dunn	5,24e-10	****
Wilcoxon	5,25e-10	****
Anova	2,1e-06	***

The cells with or without acetate were irradiated with 300 μmol photons m^–2^ s^–1^ and samples were taken at different time points. Metabolites were quantified per LC-MS/MS after quenching and extraction. ****p < 0.001, ***p < 1e-06.

Using the LC-MS/MS approach, five metabolites could not be quantified: 3-phosphoglyceric acid (3-PGA), 2-phosphoglycolate (2PG), shikimate, fructose 1,6-bisphosphate (FBP) and sedoheptulose 1,7-bisphosphate (SBP). Additionally performed enzymatic assays for 3-PGA were also negative. We consider that these five metabolites were accumulated to non-detectable levels or that they were absent because they were previously measured in wild-type Chlamydomonas using the same techniques (Mettler et al., 2014).

### Effect of Acetate on Central Carbon Metabolism and Response to Light

The CBB cycle can be divided in three parts: carboxylation and reduction followed by regeneration. In wild-type cells, the carboxylation step is catalyzed by Rubisco between CO_2_ and the five carbon ribulose-1,5-bisphosphate (RuBP), to make two three-carbon compounds (3-PGA) which is then followed by reduction to glyceraldehyde 3-phosphate. The regeneration of RuBP by phosphoribulokinase is a critical step that requires ATP and enables the system to prepare for the carboxylation reaction. As the CBB is broken in Δ*rbcL*, we quantified metabolites from different steps of the regeneration phase to comprehend to what extent the other enzymatic reactions of the CBB were still active and consuming ATP.

**FIGURE 4:**
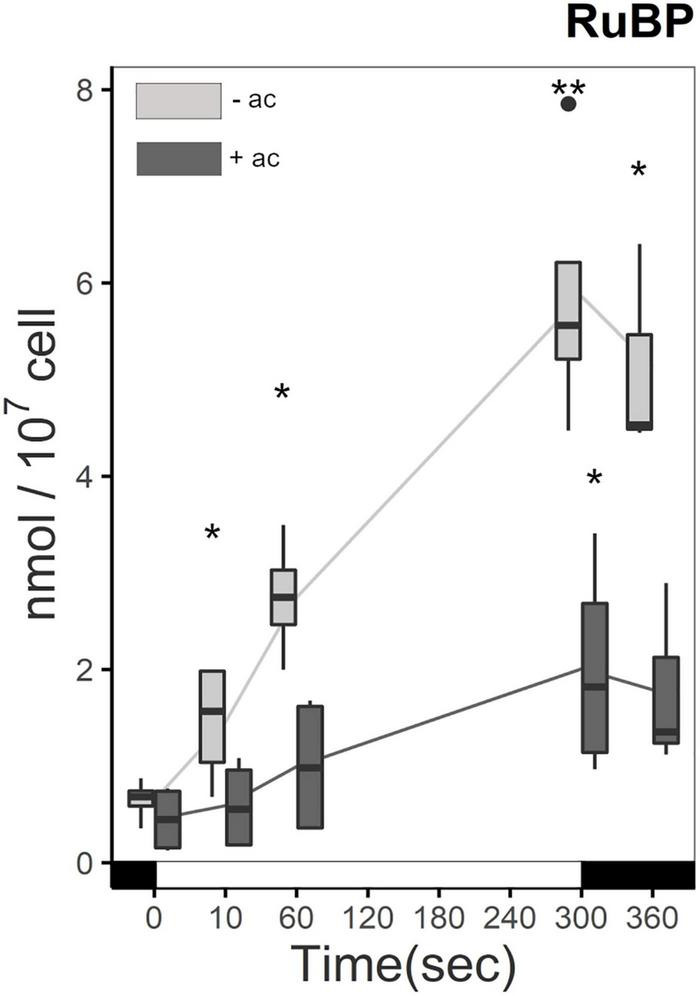
Increase in RuBP quantity in Δ*rbcL* under illumination with (dark gray) and without (light gray) acetate. Cells were irradiated with 300 μmol photons m^–2^ s^–1^ (white segment) and samples were taken at different time points (as described in Figure 2). Metabolites were quantified per LC-MS/MS after quenching and extraction. Light significantly affected the accumulation of RuBP (*p*-value < 0.001), and there was a significant difference due to acetate (*p*-value < 0.001). Pairwise *t*-test were performed between time points (one asterisk, *p* < 0.05; two asterisks, *p* < 0.01). The boxplot has been built with four replicates. The line represents the mean. The dots represent outlier data that has been taken into account for standard error and statistics, designated as a dot in the whisker plot.

Ribulose1,5-bisphosphate (RuBP) accumulation is influenced by carbon status and light in Δ*rbcL.* Before the light treatment, the amount of RuBP is similar in cells grown in medium with or without acetate. Upon light treatment and in the absence of acetate, Δ*rbcL* steadily accumulates RuBP, because it is not used in the carboxylation step normally catalyzed by RuBisCO (Figure 4). From 10 s after the beginning of the illumination RuBP accumulation between the two conditions is significantly different, reaching a four-fold greater quantity of RuBP in acetate free medium after 5 min. RuBP levels are 1–2 orders of magnitude higher than the other CBB cycle metabolites showing that RuBP is efficiently produced by the regeneration step. When returned to the dark, the RuBP quantity stabilized in both conditions. Without the carboxylation and oxygenation reaction of RuBisCO on RuBP, no 3-PGA or 2-PG are formed. The high accumulation of RuBP shows that RuBP is an endpoint in Δ*rbcL* and that these metabolites are truly absent.

**FIGURE 5:**
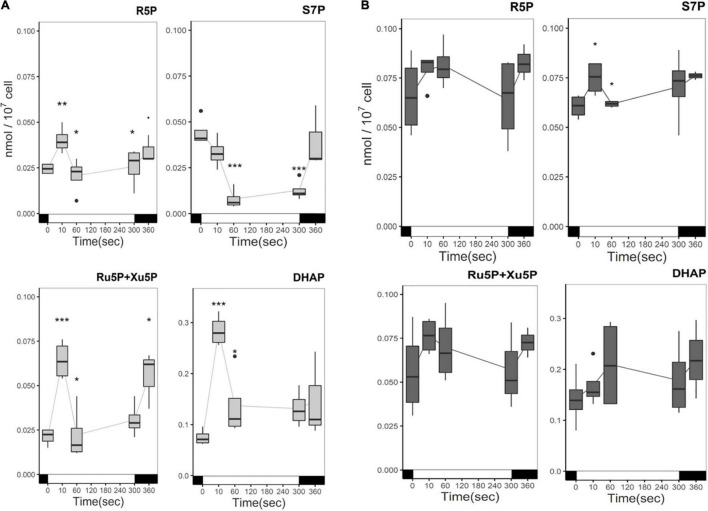
Time course of CBB intermediates metabolites in the absence **(A)** or presence **(B)** of acetate under illumination. Cells were irradiated with 300 μmol photons m^–2^ s^–1^ (white segment) and samples were taken at different time points. In the absence of acetate, light significantly affected the accumulation of CBB intermediates (*p*-value < 0.05). Pairwise *t*-test were performed between time points (one asterisk, *p* < 0.05; two asterisks, *p* < 0.01; three asterisks, *p* < 0.001). Light had no significant effect in the presence of acetate. The boxplot has been built with four replicates. The line represents the mean. The dots represent outlier data that has been taken into account for standard error and statistics, designated as a dot in the whisker plot.

The content of other CBB cycle intermediates involved in the regeneration phase is also influenced by carbon status and illumination in Δ*rbcL.* One of these, dihydroxyacetone phosphate (DHAP), is also a product of glycolysis, from the cleavage of FBP. In the absence of acetate, light has a significant effect and gives similar kinetics for: ribose 5-phosphate (R5P), sedoheptulose 7-phosphate (S7P), ribulose 5-phosphate + xylulose 5-phosphate (Ru5P + Xu5P), DHAP (Figure 5). In the first 10 s of illumination, their steady-state levels show a significant increase followed by a decrease, due to their incorporation into the metabolic flow. R5P does not change significantly from 60 s to 5 min in the light while Ru5P + Xu5P content increases. For Ru5P + Xu5P, like DHAP the content in the first 10 s greatly increases by approximatively 2–3 fold. Afterward Ru5P + Xu5P are consumed, and their content increase after a return of the cells to the dark. DHAP accumulates four times more than the other CBB cycle metabolites quantified here. S7P is the only metabolite that is directly consumed under illumination, as we observe a decrease in the first 10 s, and a significant five-fold decrease after 1 min of illumination. S7P is then regenerated in the dark, with a further three-fold increase after the dark minute. In the presence of acetate, there are no significant changes in the CBB cycle intermediates quantities in response to the light treatment. However, their quantities are significantly greater, except in the case of RuBP and DHAP, and they showed more variability between replicates than in acetate free medium (Figure 5). FBP and SBP were not detected in our analysis due to their rapid consumption. These are non-reversible reactions of the CBB cycle and considerable variations in their content have been observed between species (Arrivault et al., 2019).

**FIGURE 6:**
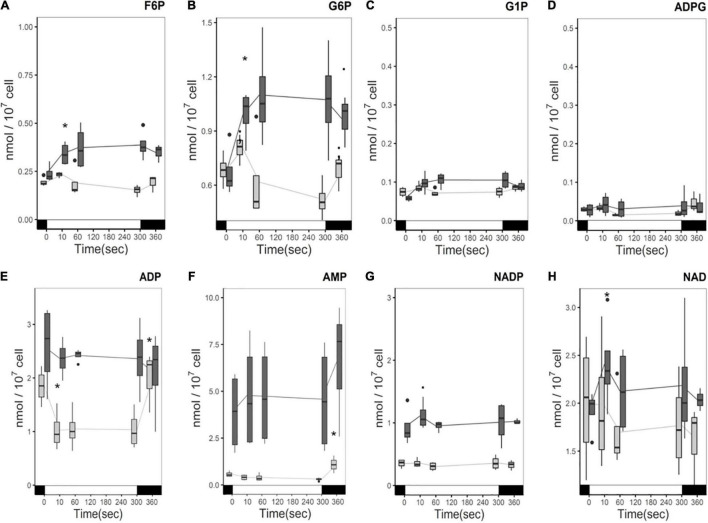
Time course of phosphorylated pentose and hexose levels **(A)** F6P, **(B)** G6P, **(C)** G1P, **(D)** ADPG, and energy precursors **(E)** ADP, **(F)** AMP, **(G)** NADP, **(H)** NAD under illumination with (dark gray) and without (light gray) acetate. Cells were illuminated with 300 μmol photons m^–2^ s^–1^ (white segment) and samples were taken at different time points. The boxplot has been built with four replicates. The line represents the mean. The dots represent outlier data that has been taken into account for standard error and statistics, designated as a dot in the whisker plot. Pairwise *t*-test were performed between time points (one square, p < 0,1; one asterisk, *p* < 0.05; two asterisks, *p* < 0.01). Light has a significant effect on the accumulation of F6P, G6P, and ADP (*p*-value < 0.05), and AMP (*p*-value < 0.01) in the absence of acetate. No significant effect of either acetate or light was found on G1P or ADPG.

The carbon status of the medium induces changes in central carbon metabolism, specifically in glycolysis and gluconeogenesis, and directionality of these pathways are directly regulated by the NADPH and ATP status of the chloroplast. Exploring those pathways would enable us to have a better appreciation of the cell’s energetic metabolism. The quantities of Fructose 6-Phosphate (F6P) and Glucose 6-Phosphate (G6P), common intermediates of glycolysis, gluconeogenesis and the oxidative pentose phosphate pathway (OPPP), showed a similar response to light: in the presence of acetate they both accumulate during the light treatment, while a decrease was observed in medium without acetate (Figures 6A,B) like for the CBB cycle intermediates. Overall, G6P accumulates three times more than F6P independently of the carbon status. Interconversion between F6P and G6P is reversible and plays an important role in regulation of the chloroplast-compartmentalized glycolytic pathway. Quantities of starch precursors, Glucose 1-Phosphate (G1P) and ADP-Glucose (ADPG) do not show significant changes in the light and accumulate 10 times less than G6P and F6P (Figures 6C,D).

Direct measures of levels of AMP, ADP, and nicotinamide adenine dinucleotide phosphate (NADP) were also undertaken to make hypotheses about the influence of the presence of acetate on AMP/ADP/ATP and NADP/NADPH levels and their response to light. AMP and ADP levels were higher in the presence of acetate (Figure 6E) compared to acetate-free medium. ADP consumption is observed in the light in the absence of acetate and ADP levels return to the same level in the dark. The ADP decrease is attributed to the production of ATP. However, in the presence of acetate no significant changes in ADP were observed over the course of the light treatment, although considerable variability was observed between samples (Figure 6E). In the presence of acetate, the AMP level is stable in the light, and its quantity more than tripled (Figure 6F). In the absence of acetate, AMP levels also do not change in the light but significantly increase after the light incubation (Figure 6F).

NADP is mostly compartmentalized in the chloroplast, while NAD is more abundant in mitochondrial and cytosolic compartments (Heber and Santarius, 1965). It has been reported that NAD is more abundant than NADP in *C. reinhardtii* (Xue et al., 1996) and we confirm this result here (Figures 6G,H). However, NADP accumulated twice as much when acetate was present than when it was absent. Nevertheless, whether this represents a higher NADP/NADPH ratio or a larger pool of NADP in response to acetate cannot be confirmed here. In contrast to ADP, where a distinct change is observed in the light, no significant changes in the light for NADP or NAD in either condition were observed (Figure 6). It should be noted that the NAD content showed a much higher degree of variation in both conditions than NADP.

**FIGURE 7:**
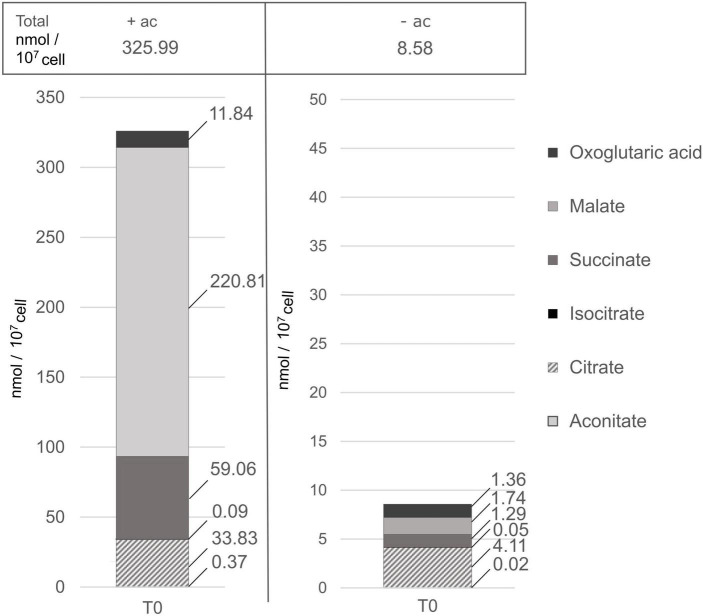
Piled histograms showing the composition in TCA and glyoxylate cycle intermediates (in nmol/10^7^cell), grown with acetate (+ac) or without (–ac) before the light treatment. 2OG, oxoglutaric acid; MAL, malate; SUCC, succinate; ICIT, isocitrate; CIT, citrate; ACON, aconitate. There are four replicates per metabolite per medium. Acetate has a significant influence on TCA and glyoxylate intermediates accumulation (*p*-value < 0.001), specifically on malate and succinate accumulation (*p*-value < 1.4 × 10^–7^).

Growth on acetate is supported by the glyoxylate cycle (Kornberg and Krebs, 1957), and we wanted to analyze the activity of this pathway in the presence or absence of acetate. This cycle is composed of five enzymes (citrate synthase, aconitase, malate dehydrogenase, isocitrate lyase, and malate synthase), the first three enzymes also participating in the TCA cycle. In *C. reinhardtii*, these two pathways are separately compartmentalized, the glyoxylate cycle is located in the cytosol or microbodies, while the TCA cycle is compartmentalized in the mitochondria (Lauersen et al., 2016). Contents of representative C4 and C6 organic acids from the TCA and glyoxylate cycles are presented in Figure 7 and as no effect of light was observed in either condition, only their content measured in the dark is displayed. These intermediates differ by more than an order of magnitude depending on the carbon status. Such variation can be explained by the activation of the glyoxylate cycle in the presence of acetate. Cells grown without acetate have a higher representation of citrate compared to the other metabolites involved in both glyoxylate and the TCA cycle. Comparing the accumulation of these metabolites in cells grown with or without acetate, the only metabolite that is not at least 10 times more accumulated is isocitrate. In the presence of acetate, on average succinate accumulates 50 times more and malate accumulates 100 times more than the other organic acids. The biosynthesis of some amino acids is also linked to the TCA cycle. The content of glutamate and aspartate that use oxoglutaric acid and oxaloacetate as precursors for their synthesis accumulated to 10 times greater quantities in cells grown in acetate compared to acetate-free medium (*p-*value < 5.5 × 10^–7^) (Supplementary Table 3).

## Discussion

### Acetate Assimilation in Δ*rbcL* Results in Cooperation of Tricarboxylic Acid and Glyoxylate Cycles, Inhibition of Calvin Benson Bassham Cycle, Upregulation of Oxidative Pentose Phosphate Pathway, and Limits ATP Production in the Chloroplast

Optimal functioning of photosynthesis depends on the balance between NADPH and ATP. During mixotrophic growth, acetate assimilation changes the balance, consuming ATP and producing reducing equivalents (Johnson and Alric, 2013). This results in rerouting of electrons from LEF to CEF involving only PSI to generate ATP, but not NADPH (Johnson and Alric, 2012; Lucker and Kramer, 2013). In the absence of an active CBB cycle, this imbalance is amplified, making Δ*rbcL* a good system for studying the energetic balance required for photosynthetic electron transport and metabolic reactions other than CO_2_ assimilation. Here we show that acetate assimilation causes a significant rerouting of metabolites in Δ*rbcL* and this has effects on the regulation of the photosynthetic electron transport chain.

**FIGURE 8:**
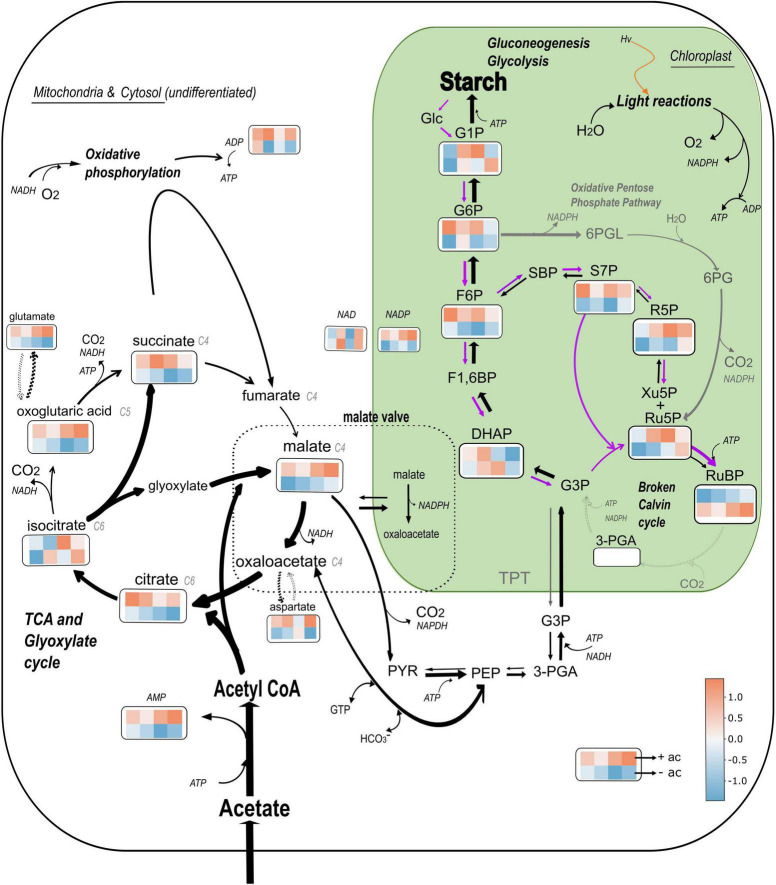
Model of central C metabolism in *Chlamydomonas reinhardtii* Δ*rbcL* mutant depending on the carbon status. The relative changes of the abundance of 19 metabolites is represented using colored squares extracted from a heatmap. The top four squares are replicates that represent metabolite abundance in presence of acetate, and the bottom four in absence of acetate, both after a 5 min light treatment (300 μmol photons m^–2^ s^–1^). The major pathways shown are the glyoxylate and TCA cycles represented together, the gluconeogenic pathway from PYR and PEP to Starch, the Oxidative Pentose Phosphate pathway (gray arrows) and the Calvin Benson Bassham cycle, represented here as “broken” due to the absence of RuBisCO (dotted gray arrows). The width of the black arrows represents the preferential flows in heterotrophic conditions. Purple arrows represents preferential flows in absence of acetate. Glc, glucose; GTP, Guanosine-5’-triphosphate; 6PGL, 6-phosphogluconolactone; 6PG, 6-phosphogluconic acid. Data has been normalized by metabolite and heatmaps have been generated by the heatmaply package on Rstudio. Color is based on correlation coefficient according to the color scale indicated.

The model presented in Figure 8 shows a proposition for the metabolic flows in the presence of acetate (black and gray arrows) and without acetate (purple arrows) with a heatmap for each of the metabolites quantified. The heatmaps illustrate that Δ*rbcL* cells grown in acetate medium are metabolically active and produce higher quantities of most of the metabolites with significant amino acid accumulation (see aspartate and glutamate accumulation in Supplementary Table 3).

Mutants that do not accumulate RuBisCo do not have a functional pyrenoid (Rawat et al., 1996) so differences that have been identified in this study are due to the upregulation of heterotrophic metabolism in the presence of acetate. These substantial differences can be understood as survival strategies: in the presence of acetate, the maintenance of glyoxylate and TCA cycles for the storage of starch reserves becomes vital and this drives a movement of reductant (NADPH and gluconeogenic precursors) toward the chloroplast that represses both photosynthetic electron flow and the CBB cycle. In the absence of acetate, Δ*rbcL* cells are carbon ‘starved’ and use the light and the breakdown of starch reserves to drive forward ATP consuming reactions in the chloroplast, including the “broken” CBB cycle to maintain photosynthetic electron flow to the detriment of respiration.

#### Acetate Assimilation Results in Cooperation of Tricarboxylic Acid and Glyoxylate Cycle

The major pathway for acetate assimilation is its conversion to acetyl coenzyme A (acetyl-CoA), which subsequently feeds the glyoxylate cycle. The net product of this cycle is succinate providing carbon skeletons and reducing equivalents to drive metabolism (Boyle and Morgan, 2009; Roach et al., 2013; Chapman et al., 2015; Boyle et al., 2017). Acetate assimilation requires ATP to facilitate active uptake and for the formation of acetyl-CoA, occurring along two pathways: *via* AMP-forming acetyl CoA synthase (ACS) or *via* a pathway involving acetate kinase (ACK) and phosphate acetyltransferase (PAT) (Johnson and Alric, 2013; Chapman et al., 2015). AMP is highly accumulated and the ACS pathway would be an important source.

Acetyl-CoA (C2) and oxaloacetate (C4) produce citrate that is converted into isocitrate. Due to the ratios of accumulated C4 and C6 organic acids we adopt the non-compartmentalized models [according to Boyle et al. (2017) for heterotrophic growth and Chapman et al. (2015) for mixotrophic growth], that are combined to more simply illustrate the cooperation of TCA and glyoxylate cycles. The product of these pathways is an accumulation of organic and amino acids (aspartate and glutamate, see Supplementary Table 3). From isocitrate, most of the flux is predicted to be *via* isocitrate lyase (ICL), which converts isocitrate into glyoxylate and succinate and ensures that the CO_2_ evolution steps of the cycle are bypassed, resulting in retention of organic carbon by the cell, which is essential for efficient mixotrophic growth (Plancke et al., 2014). Thus, glyoxylate cycle surpasses TCA cycle activity in Δ*rbcL* witnessed by a significant succinate accumulation, the by-product of the glyoxylate cycle, and the surplus is not fully metabolized by the TCA cycle (Figures 7, 8).

#### Anaplerotic Pathway Maintains ATP Production by Respiration in the Presence of Acetate

Our results suggest an activity of the anaplerotic pathway that regenerates TCA cycle intermediates (specifically oxaloacetate and oxoglutaric acid) to curb the leak of these intermediates toward aspartate and glutamate synthesis (Norici and Giordano, 2002). Beta-carboxylations that produce oxaloacetate from phosphoenolpyruvate (PEP) in green algae are induced by high ATP levels and require a net uptake of HCO_3_^–^ (Treves et al., 2021). Under conditions when CO_2_ fixation is compromised, significant levels of HCO_3_^–^ uptake have been measured by stimulating glutamate synthesis in *C. reinhardtii*, as well as in the green alga *Selenastrum minutum* (Guy et al., 1989; Giordano et al., 2003). In our case, a 100 fold increase in malate content may be indicating a significant contribution from this pathway. From an energetic perspective, by maintaining the TCA cycle, the anaplerotic pathway also maintains ATP production by respiration. This results in consequences for ATP production by photosynthesis in the chloroplast because the limited pool of ADP will be preferentially used by the mitochondria. In wild-type cells, total ADP and AMP levels rise in light relative to ATP levels in *C. reinhardtii* (Xue et al., 1996), making the compartmentalization of ADP an important factor to maintain ATP synthesis or when ADP shortages do occur to stimulate photoprotective pathways (Takizawa et al., 2008). Free inorganic phosphate (Pi), ADP and ATP limitation, and their consequences on metabolism and alternative electron transfer pathways in the chloroplast are further discussed below.

#### Acetate Assimilation Leads to an Import of Gluconeogenic Precursors Into the Chloroplast and Starch Accumulation

Starch can be formed from oxaloacetate and malate that are respectively converted to PEP and pyruvate, precursor compounds of gluconeogenesis (Chapman et al., 2015). Malate is also used for redox communication between compartments, *via* the malate valve to balance ATP/NAD(P)H ratio (Xue et al., 1996; Scheibe, 2004). The NADP-Malate Dehydrogenase reversibly reduces oxaloacetate to malate and oxidizes NADPH. Orthologs of known 2-oxoglutarate/malate transporters (Weber et al., 1995) are found in the *C. reinhardtii* genome and one of them is found in the chloroplast proteome (Terashima et al., 2011) and is significantly upregulated at the transcriptional level under mixotrophic conditions (Puzanskiy et al., 2020). The malate shunt operates in two directions, excess NADPH is consumed in the mitochondria to equilibrate the NADPH/NADP + ratio, or NADPH is supplied to the chloroplast by the mitochondria in heterotrophic/mixotrophic conditions (Boyle and Morgan, 2009; Chapman et al., 2015). The highly reduced photosynthetic chain observed in Figure 2 in the presence of acetate lends support to an accumulation of reducing power in the chloroplast, in part attributable to the malate shuttle, leading to a strong accumulation of malate as this compartment becomes increasingly reduced (Figure 8). While we did not witness a decrease in NADP levels (Figure 6G), this is because pools of NADP do not strictly represent the NADPH/NADP ratio.

#### The Disrupted Calvin Benson Bassham Cycle Results in a Limited Response to Light Caused by Chloroplastic Reducing Power Imbalance and Pi Limitation in the Presence of Acetate

With acetate as a carbon source, Δ*rbcL* metabolites showed no or little response to light. During a light induction, the *sta6* mutant that has active photosynthetic electron transfer and an intact CBB cycle but no starch accumulation, shows a decreased photosynthetic activity compared to the wild-type under mixotrophic conditions with a slow rise in chlorophyll fluorescence. Krishnan et al. (2015) linked it to a reduced plastoquinone pool and a slowing down of NADPH re-oxidation, hence a build-up in the reductant poise (NADPH/NADP+), and an accumulation of CBB cycle and gluconeogenic precursors. Although we observed significant starch accumulation in Δ*rbcL*, the disrupted CBB cycle also results in a NADPH/NADP + imbalance due to the absence of the reduction phase and the strong import of gluconeogenic precursors.

The sequestration of Pi into phosphorylated hexose and pentose would eventually result, as suggested in the *sta6* mutant, in a decrease of Pi in the stroma of the chloroplast and increase of the proton motive force (*pmf) via* regulation of the ATP synthase and PGR5 (Fisher et al., 2019). Our results show that little ADP consumption was witnessed in the light. However, as respiration is stimulated in the presence of acetate, only small changes in net whole cell ADP levels would be expected due to ongoing oxidative phosphorylation. Nevertheless, CEF activity in the presence of acetate was expected to drive a considerable ATP production. Here, ATP synthesis may be restricted due to Pi limitation despite the low luminal pH created by CEF. Low luminal pH gives rise to photosynthetic control that we witness by the slowing down of total electron flow.

#### Gluconeogenesis Cooperates With Oxidative Pentose Phosphate Pathway and the Broken Calvin Benson Bassham Cycle to Efficiently Accumulate Starch in Δ*rbcL*

The only metabolites that show a significant accumulation in response to light are G6P and F6P and we attribute this to the OPPP. In the oxidative branch, G6P is converted to Ru5P and CO_2_, with the reduction of two NADP to NADPH. In the non-oxidative branch of the OPPP, three molecules of Ru5P are converted to two molecules of F6P and one molecule of GAP (Stincone et al., 2015; Sharkey, 2021). The oxidative OPPP in the chloroplast is inhibited in the light by reduction of two cysteine residues of a stromal G6P dehydrogenase (G6PDH) *via* the thioredoxin system. However, this inhibition which prevents the G6P shunt can be overcome by high concentrations of G6P or H_2_O_2_ (Wenderoth et al., 1997; Preiser et al., 2019), both indicators of over-reduction at the level of PSI. Entry of G6P will result in ATP consumption and stimulation of cyclic electron flow, to relieve the excess pressure at PSI. Stimulation of cyclic electron flow by H_2_O_2_ has also been reported (Strand et al., 2015). Flux balance analysis predictions show that in the presence of acetate, OPPP activity is increased, channeling photosynthates into nucleotide synthesis, as carbon fixation in the CBB cycle is reduced (Chapman et al., 2015, 2017). Within the chloroplast compartment, we propose that gluconeogenesis cooperates with OPPP and the broken CBB cycle to accumulate starch efficiently in Δ*rbcL* (Figure 8).

### Incubation of Δ*rbcL* Without Acetate Results in a Light Activated Carbon Metabolism

The high starch reserves accumulated in Δ*rbcL* allow its survival in the absence of an exogenous carbon source for a number of days (this work and Johnson et al., 2010) allowing us to test acetate versus non-acetate culture conditions. Despite the absence of RuBisCO and a non-functional carbon-fixation step, other steps of the CBB cycle remained active in the light. This is observed as a transient accumulation followed by a consumption of R5P, Ru5P + Xu5P, and DHAP. Rapid depletion kinetics in the light were observed for S7P produced by Sedoheptulose-1,7-Bisphosphatase (SBP), as expected for a rate limiting enzyme (Hammel et al., 2020). Metabolite measurements in *C. reinhardtii* show that only a portion of the CBB cycle enzymes are near-saturated *in vivo* [RuBisCO, Phosphoglycerate Kinase (PGK), Fructose-1,6-Bisphosphatase (FBPase), SBPase] whilst the remainder operate at low substrate saturation (Mettler et al., 2014). We propose that breakdown of starch provided the input of phosphorylated hexose *via* glycolysis to feed into RuBP accumulation. Phosphoribulokinase (PRK) activity requires ATP, and ADP pools were depleted in the light.

ATP is also consumed in the upper half of glycolysis (starch degradation) and here we observe a consumption of G6P and F6P with similar kinetics to the CBB cycle intermediates. The consumption of G6P/F6P in the light is attributable to Phosphofructokinase (PFK), which is regulated by cellular ATP levels in *C. reinhardtii* (Bulté et al., 1990). These two lines of evidence, ATP consumption by glycolysis and the production of significant quantities of RuBP, show that ADP is not limiting in the chloroplast under the acetate-deplete condition. In previous work on a RuBisCO mutant, in the absence of acetate, no CO_2_ evolution from the TCA cycle could be measured in the light (Gans and Rebeille, 1988). In our work, the very low levels of C4 and C6 organic acids with no change in accumulation in the light, are in agreement that the TCA cycle is indeed inhibited. We are in agreement with Gans and Rebeille (1988) that the best hypothesis is that this is driven by competition for cytosolic or available cellular ADP reserves. The consequence of the inhibition of respiration would be that ADP is not limiting in the chloroplast and little control is thus exerted to down regulate LEF. We propose that Δ*rbcL*, in the absence of acetate uses chloroplastic oxygen photoreduction and RuBP accumulation as acceptors for electrons, drawing ADP into the chloroplast at the expense of respiration.

## Conclusion

In wild-type cells of Chlamydomonas, at the onset of an illumination, respiration increases with light intensity and that, in turn, stimulates PSI activity (Healey and Myers, 1971). Over a similar time frame, in Arabidopsis, a light-induced alkanization of the mitochondrial matrix suggests rapid respiration stimulation and ATP synthesis (Elsässer et al., 2020). This process assumedly works *via* the malate shuttle both in plants and algae (Xue et al., 1996; Scheibe, 2004). These results suggest that respiration serves a role in energetic “priming” until the photosynthetic chain and the CBB cycle are coupled and later these interactions becomes less important if conditions remain stable. Without PGR5 activity, respiration is constitutively stimulated in both wild-type and Δ*rbcL* backgrounds in the presence or absence of acetate (Johnson et al., 2014; Steinbeck et al., 2015). PGR5 regulates CEF and increases luminal acidification that results in a down regulation of total electron flow (photosynthetic control). In the absence of RuBisCO where linear electron flow is restricted, a deletion of PGR5 (Δ*rbcL pgr5*) increases linear electron flow. Despite the similarity in chlorophyll fluorescence kinetics between Δ*rbcL* in the absence of acetate and Δ*rbcL pgr5*, the interactions between electron transport and metabolism are not the same. Our current data shows the strong remodeling of metabolism that boosts respiratory pathways in Δ*rbcL* when acetate is present. We hypothesize that Δ*rbcL* in an absence of acetate would indeed require CEF activity to recycle NADPH and drive ATP formation.

One of the aims of the present work was to identify metabolic pathways that control the regulation of CEF in *C. reinhardtii*. In the presence of acetate, we have identified malate and gluconeogenic precursors, especially G6P and F6P, as candidates for the stimulation of photosynthetic control mechanisms leading to inhibition of linear electron flow and CBB cycle. On the other hand, when acetate is absent, we show that a broken CBB cycle can maintain electron transfer, at least over a 5 min illumination, because ADP and Pi are preferentially available inside the chloroplast rather than driving respiratory pathways.

## Data Availability Statement

The original contributions presented in the study are included in the article/Supplementary Material, further inquiries can be directed to the corresponding author/s.

## Author Contributions

MS-S, PB, SA, JA, and XJ contributed to conception and design of the study. MS-S and SA organized the database. MS-S performed the statistical analysis. MS-S and XJ wrote the first draft of the manuscript. PB wrote sections of the manuscript. All authors contributed to manuscript revision, read, and approved the submitted version.

## Conflict of Interest

The authors declare that the research was conducted in the absence of any commercial or financial relationships that could be construed as a potential conflict of interest.

## Publisher’s Note

All claims expressed in this article are solely those of the authors and do not necessarily represent those of their affiliated organizations, or those of the publisher, the editors and the reviewers. Any product that may be evaluated in this article, or claim that may be made by its manufacturer, is not guaranteed or endorsed by the publisher.

## Footnotes

1
https://www.metaboanalyst.ca

## Supplementary Material

Supplementary Table 1 Twenty-three chosen metabolites and their metabolic pathways.

Supplementary Table 2 Statistic tests explanations.

Supplementary Table 3 Metabolite data.

## References

[B1] Allen J. F. de Paula W. B. M. Puthiyaveetil S. Nield J. (2011). A structural phylogenetic map for chloroplast photosynthesis. *Trends Plant Sci.* 16 645–655. 10.1016/j.tplants.2011.10.004 22093371

[B2] Alric J. Lavergne J. Rappaport F. (2010). Redox and ATP control of photosynthetic cyclic electron flow in *Chlamydomonas reinhardtii* (I) aerobic conditions. *Biochim. Biophys. Acta* 1797 44–51. 10.1016/j.bbabio.2009.07.009 19651104

[B3] Arrivault S. Alexandre Moraes T. Obata T. Medeiros D. B. Fernie A. R. Boulouis A. (2019). Metabolite profiles reveal interspecific variation in operation of the Calvin–Benson cycle in both C4 and C3 plants. *J. Exp. Bot.* 70 1843–1858. 10.1093/jxb/erz051 30773587 PMC6436152

[B4] Arrivault S. Guenther M. Fry S. C. Fuenfgeld M. M. F. F. Veyel D. Mettler-Altmann T. (2015). Synthesis and use of stable-isotope-labeled internal standards for quantification of phosphorylated metabolites by LC-MS/MS. *Anal. Chem.* 87 6896–6904. 10.1021/acs.analchem.5b01387 26010726

[B5] Arrivault S. Guenther M. Ivakov A. Feil R. Vosloh D. Van Dongen J. T. (2009). Use of reverse-phase liquid chromatography, linked to tandem mass spectrometry, to profile the Calvin cycle and other metabolic intermediates in *Arabidopsis* rosettes at different carbon dioxide concentrations. *Plant J.* 59 826–839. 10.1111/j.1365-313X.2009.03902.x 19453453

[B6] Avenson T. J. Cruz J. A. Kanazawa A. Kramer D. M. (2005). Regulating the proton budget of higher plant photosynthesis. *Proc. Natl. Acad. Sci. U.S.A.* 102 9709–9713. 10.1073/pnas.0503952102 15972806 PMC1172270

[B7] Bölling C. Fiehn O. (2005). Metabolite profiling of *Chlamydomonas reinhardtii* under nutrient deprivation. *Plant Physiol.* 139 1995–2005. 10.1104/pp.105.071589 16306140 PMC1310576

[B8] Boyle N. R. Morgan J. A. (2009). Flux balance analysis of primary metabolism in *Chlamydomonas reinhardtii*. *BMC Syst. Biol.* 3:4. 10.1186/1752-0509-3-4 19128495 PMC2628641

[B9] Boyle N. R. Sengupta N. Morgan J. A. (2017). Metabolic flux analysis of heterotrophic growth in *Chlamydomonas reinhardtii*. *PLoS One* 12:e0177292. 10.1371/journal.pone.0177292 28542252 PMC5443493

[B10] Bulté L. Gans P. Rebéillé F. Wollman F.-A. (1990). ATP control on state transitions in vivo in *Chlamydomonas reinhardtii*. *Biochim. Biophys. Acta Bioenerget.* 1020 72–80. 10.1016/0005-2728(90)90095-L

[B11] Chapman S. P. Paget C. M. Johnson G. N. Schwartz J.-M. (2015). Flux balance analysis reveals acetate metabolism modulates cyclic electron flow and alternative glycolytic pathways in *Chlamydomonas reinhardtii*. *Front. Plant Sci.* 6:474. 10.3389/fpls.2015.00474 26175742 PMC4485056

[B12] Chapman S. P. Trindade dos Santos M. Johnson G. N. Kritz M. V. Schwartz J.-M. (2017). Cyclic decomposition explains a photosynthetic down regulation for *Chlamydomonas reinhardtii*. *Biosystems* 162 119–127. 10.1016/j.biosystems.2017.09.014 28970020 PMC5720477

[B13] Chaux F. Burlacot A. Mekhalfi M. Auroy P. Blangy S. Richaud P. (2017). Flavodiiron proteins promote fast and transient O2 photoreduction in Chlamydomonas1[OPEN]. *Plant Physiol.* 174 1825–1836. 10.1104/pp.17.00421 28487478 PMC5490913

[B14] Chaux F. Peltier G. Johnson X. (2015). A security network in PSI photoprotection: regulation of photosynthetic control, NPQ and O2 photoreduction by cyclic electron flow. *Front. Plant Sci.* 6:875. 10.3389/fpls.2015.00875 26528325 PMC4606052

[B15] Dang K.-V. Plet J. Tolleter D. Jokel M. Cuiné S. Carrier P. (2014). Combined increases in mitochondrial cooperation and oxygen photoreduction compensate for deficiency in cyclic electron flow in *Chlamydomonas reinhardtii*. *Plant Cell* 26 3036–3050. 10.1105/tpc.114.126375 24989042 PMC4145130

[B16] Dumas L. Chazaux M. Peltier G. Johnson X. Alric J. (2016). Cytochrome b6f function and localization, phosphorylation state of thylakoid membrane proteins and consequences on cyclic electron flow. *Photosynthesis Res.* 129 307–320. 10.1007/s11120-016-0298-y 27534565

[B17] Elsässer M. Feitosa-Araujo E. Lichtenauer S. Wagner S. Fuchs P. Giese J. (2020). Photosynthetic activity triggers pH and NAD redox signatures across different plant cell compartments (p. 2020.10.31.363051). *bioRxiv* [preprint]. 10.1101/2020.10.31.363051v1

[B18] Fisher N. Bricker T. M. Kramer D. M. (2019). Regulation of photosynthetic cyclic electron flow pathways by adenylate status in higher plant chloroplasts. *Biochim. Biophys. Acta Bioenerget.* 1860:148081. 10.1016/j.bbabio.2019.148081 31520615

[B19] Gans P. Rebeille F. (1988). Light inhibition of mitochondrial respiration in a mutant of *Chlamydomonas reinhardtii* devoid of ribulose-1,5-bisphosphate carboxylase/oxygenase activity. *Arch. Biochem. Biophys.* 260 109–117. 10.1016/0003-9861(88)90430-4 3341736

[B20] Gibbs M. Gfeller R. P. Chen C. (1986). Fermentative metabolism of *Chlamydomonas reinhardii*1. *Plant Physiol.* 82 160–166. 10.1104/pp.82.1.160 16664985 PMC1056083

[B21] Giordano M. Norici A. Gilmour D. J. (2003). Influence of the nitrogen source and metabolites on the Vmax of phosphoenolpyruvate carboxylase from the unicellular green alga *Dunaliella parva* CCAP 19/9 (Volvocales, Chlorophyceae). *Phycologia* 42 133–137. 10.2216/i0031-8884-42-2-133.1

[B22] Guy R. D. Vanlerberghe G. C. Turpin D. H. (1989). Significance of phosphoenolpyruvate carboxylase during ammonium assimilation: carbon isotope discrimination in photosynthesis and respiration by the N-limited green alga *Selenastrum minutum* 1. *Plant Physiol.* 89 1150–1157. 10.1104/pp.89.4.1150 16666678 PMC1055989

[B23] Hammel A. Sommer F. Zimmer D. Stitt M. Mühlhaus T. Schroda M. (2020). Overexpression of sedoheptulose-1,7-bisphosphatase enhances photosynthesis in *Chlamydomonas reinhardtii* and has no effect on the abundance of other calvin-benson cycle enzymes. *Front. Plant Sci.* 11:868. 10.3389/fpls.2020.00868 32655601 PMC7324757

[B24] Healey F. P. Myers J. (1971). The Kok Effect in *Chlamydomonas reinhardi*. *Plant Physiol.* 47 373–379. 10.1104/pp.47.3.373 16657625 PMC365872

[B25] Heber U. W. Santarius K. A. (1965). Compartmentation and reduction of pyridine nucleotides in relation to photosynthesis. *Biochim. Biophys. Acta* 109 390–408. 10.1016/0926-6585(65)90166-4 4379647

[B26] Heifetz P. B. Förster B. Osmond C. B. Giles L. J. Boynton J. E. (2000). Effects of acetate on facultative autotrophy in *Chlamydomonas reinhardtii* assessed by photosynthetic measurements and stable isotope analyses. *Plant Physiol.* 122 1439–1445. 10.1104/pp.122.4.1439 10759539 PMC58978

[B27] Johnson X. (2011). Manipulating RuBisCO accumulation in the green alga, *Chlamydomonas reinhardtii*. *Plant Mol. Biol.* 76 397–405. 10.1007/s11103-011-9783-z 21607658

[B28] Johnson X. Alric J. (2012). Interaction between starch breakdown, acetate assimilation, and photosynthetic cyclic electron flow in *Chlamydomonas reinhardtii*. *J. Biol. Chem.* 287 26445–26452. 10.1074/jbc.M112.370205 22692199 PMC3406727

[B29] Johnson X. Alric J. (2013). Central carbon metabolism and electron transport in *Chlamydomonas reinhardtii*: metabolic constraints for carbon partitioning between oil and starch. *Eukaryotic Cell* 12 776–793. 10.1128/EC.00318-12 23543671 PMC3675994

[B30] Johnson X. Steinbeck J. Dent R. M. Takahashi H. Richaud P. Ozawa S.-I. (2014). Proton gradient regulation 5-mediated cyclic electron flow under ATP- or redox-limited conditions: a study of Δ ATPase pgr5 and Δ rbcL pgr5 mutants in the green alga *Chlamydomonas reinhardtii*. *Plant Physiol.* 165 438–452. 10.1104/pp.113.233593 24623849 PMC4012601

[B31] Johnson X. Wostrikoff K. Finazzi G. Kuras R. Schwarz C. Bujaldon S. (2010). MRL1, a conserved pentatricopeptide repeat protein, is required for stabilization of rbcL mRNA in *Chlamydomonas* and *Arabidopsis*. *Plant Cell* 22 234–248. 10.1105/tpc.109.066266 20097872 PMC2828700

[B32] Joliot P. Joliot A. (2002). Cyclic electron transfer in plant leaf. *Proc. Natl. Acad. Sci. U.S.A.* 99 10209–10214. 10.1073/pnas.102306999 12119384 PMC126649

[B33] Kempa S. Hummel J. Schwemmer T. Pietzke M. Strehmel N. Wienkoop S. (2009). An automated GCxGC-TOF-MS protocol for batch-wise extraction and alignment of mass isotopomer matrixes from differential 13C-labelling experiments: a case study for photoautotrophic-mixotrophic grown *Chlamydomonas reinhardtii* cells. *J. Basic Microbiol.* 49 82–91. 10.1002/jobm.200800337 19206143

[B34] Klein U. Betz A. (1978). Fermentative metabolism of hydrogen-evolving *Chlamydomonas moewusii*. *Plant Physiol.* 61 953–956. 10.1104/pp.61.6.953 16660433 PMC1092019

[B35] Kornberg H. L. Krebs H. A. (1957). Synthesis of cell constituents from C2-units by a modified tricarboxylic acid cycle. *Nature* 179 988–991. 10.1038/179988a0 13430766

[B36] Krishnan A. Kumaraswamy G. K. Vinyard D. J. Gu H. Ananyev G. Posewitz M. C. (2015). Metabolic and photosynthetic consequences of blocking starch biosynthesis in the green alga *Chlamydomonas reinhardtii* sta6 mutant. *Plant J.* 81 947–960. 10.1111/tpj.12783 25645872

[B37] Lauersen K. J. Baier T. Wichmann J. Wördenweber R. Mussgnug J. H. Hübner W. (2016). Efficient phototrophic production of a high-value sesquiterpenoid from the eukaryotic microalga *Chlamydomonas reinhardtii*. *Metab. Eng.* 38 331–343. 10.1016/j.ymben.2016.07.013 27474353

[B38] Lucker B. Kramer D. M. (2013). Regulation of cyclic electron flow in *Chlamydomonas reinhardtii* under fluctuating carbon availability. *Photosynthesis Res.* 117 449–459. 10.1007/s11120-013-9932-0 24113925

[B39] Lunn J. E. Feil R. Hendriks J. H. M. Gibon Y. Morcuende R. Osuna D. (2006). Sugar-induced increases in trehalose 6-phosphate are correlated with redox activation of ADPglucose pyrophosphorylase and higher rates of starch synthesis in *Arabidopsis thaliana*. *Biochem. J.* 397 139–148. 10.1042/BJ20060083 16551270 PMC1479759

[B40] Mathiot C. Alric J. (2021). Standard units for ElectroChromic Shift measurements in plant biology. *J. Exp. Bot.* 72 6467–6473. 10.1093/jxb/erab261 34089606

[B41] Mettler T. Mühlhaus T. Hemme D. Schöttler M.-A. Rupprecht J. Idoine A. (2014). Systems analysis of the response of photosynthesis, metabolism, and growth to an increase in irradiance in the photosynthetic model organism *Chlamydomonas reinhardtii*. *Plant Cell* 26 2310–2350. 10.1105/tpc.114.124537 24894045 PMC4114937

[B42] Norici A. Giordano M. (2002). Anaplerosis in microalgae. *Recent Res. Dev. Plant Physiol.* 3 153–164. 10.1034/j.1399-3054.2002.1160207.x 12354194

[B43] Plancke C. Vigeolas H. Höhner R. Roberty S. Emonds-Alt B. Larosa V. (2014). Lack of isocitrate lyase in *Chlamydomonas* leads to changes in carbon metabolism and in the response to oxidative stress under mixotrophic growth. *Plant J.* 77 404–417. 10.1111/tpj.12392 24286363

[B44] Porra R. J. Thompson W. A. Kriedemann P. E. (1989). Determination of accurate extinction coefficients and simultaneous equations for assaying chlorophylls a and b extracted with four different solvents: verification of the concentration of chlorophyll standards by atomic absorption spectroscopy. *Biochim. Biophys. Acta Bioenerget.* 975 384–394. 10.1016/S0005-2728(89)80347-0

[B45] Preiser A. L. Fisher N. Banerjee A. Sharkey T. D. (2019). Plastidic glucose-6-phosphate dehydrogenases are regulated to maintain activity in the light. *Biochem. J.* 476 1539–1551. 10.1042/BCJ20190234 31092702 PMC6626494

[B46] Puzanskiy R. K. Romanyuk D. A. Shishova M. F. (2020). Shift in expression of the genes of primary metabolism and chloroplast transporters in *Chlamydomonas reinhardtii* under different trophic conditions. *Russian J. Plant Physiol.* 67 867–878. 10.1134/S102144372005012X

[B47] Rawat M. Henk M. C. Lavigne L. L. Moroney J. V. (1996). *Chlamydomonas reinhardtii* mutants without ribulose-1,5-bisphosphate carboxylase-oxygenase lack a detectable pyrenoid. *Planta* 198 263–270. 10.1007/BF00206252

[B48] Roach T. Sedoud A. Krieger-Liszkay A. (2013). Acetate in mixotrophic growth medium affects photosystem II in *Chlamydomonas reinhardtii* and protects against photoinhibition. *Biochim. Biophys. Acta* 1827 1183–1190. 10.1016/j.bbabio.2013.06.004 23791666

[B49] Sasso S. Stibor H. Mittag M. Grossman A. R. (2018). From molecular manipulation of domesticated *Chlamydomonas reinhardtii* to survival in nature. *eLife* 7:e39233. 10.7554/eLife.39233 30382941 PMC6211829

[B50] Scheibe R. (2004). Malate valves to balance cellular energy supply. *Physiol. Plant.* 120 21–26. 10.1111/j.0031-9317.2004.0222.x 15032873

[B51] Scoma A. Giannelli L. Faraloni C. Torzillo G. (2012). Outdoor H2 production in a 50-L tubular photobioreactor by means of a sulfur-deprived culture of the microalga *Chlamydomonas reinhardtii*. *J. Biotechnol.* 157 620–627. 10.1016/j.jbiotec.2011.06.040 21771618

[B52] Sharkey T. D. (2021). Pentose phosphate pathway reactions in photosynthesizing cells. *Cells* 10:1547. 10.3390/cells10061547 34207480 PMC8234502

[B53] Steinbeck J. Nikolova D. Weingarten R. Johnson X. Richaud P. Peltier G. (2015). Deletion of Proton Gradient Regulation 5 (PGR5) and PGR5-Like 1 (PGRL1) proteins promote sustainable light-driven hydrogen production in *Chlamydomonas reinhardtii* due to increased PSII activity under sulfur deprivation. *Front. Plant Sci.* 6:892. 10.3389/fpls.2015.00892 26579146 PMC4621405

[B54] Stincone A. Prigione A. Cramer T. Wamelink M. M. C. Campbell K. Cheung E. (2015). The return of metabolism: biochemistry and physiology of the pentose phosphate pathway. *Biol. Rev. Cambr. Philos. Soc.* 90 927–963. 10.1111/brv.12140 25243985 PMC4470864

[B55] Strand D. D. Fisher N. Davis G. A. Kramer D. M. (2016). Redox regulation of the antimycin A sensitive pathway of cyclic electron flow around photosystem I in higher plant thylakoids. *Biochim. Biophys. Acta Bioenerget.* 1857 1–6. 10.1016/j.bbabio.2015.07.012 26235611

[B56] Strand D. D. Livingston A. K. Satoh-Cruz M. Froehlich J. E. Maurino V. G. Kramer D. M. (2015). Activation of cyclic electron flow by hydrogen peroxide in vivo. *Proc. Natl. Acad. Sci. U.S.A.* 112 5539–5544. 10.1073/pnas.1418223112 25870290 PMC4418880

[B57] Suorsa M. Järvi S. Grieco M. Nurmi M. Pietrzykowska M. Rantala M. (2012). PROTON GRADIENT REGULATION5 is essential for proper acclimation of *Arabidopsis* photosystem I to naturally and artificially fluctuating light conditions. *Plant Cell* 24 2934–2948. 10.1105/tpc.112.097162 22822205 PMC3426124

[B58] Takahashi H. Clowez S. Wollman F.-A. Vallon O. Rappaport F. (2013). Cyclic electron flow is redox-controlled but independent of state transition. *Nat. Commun.* 4:1954. 10.1038/ncomms2954 23760547 PMC3709502

[B59] Takizawa K. Kanazawa A. Kramer D. M. (2008). Depletion of stromal Pi induces high ‘energy-dependent’ antenna exciton quenching (qE) by decreasing proton conductivity at CFO-CF1 ATP synthase. *Plant Cell Environ.* 31 235–243. 10.1111/j.1365-3040.2007.01753.x 17996016

[B60] Terashima M. Specht M. Hippler M. (2011). The chloroplast proteome: a survey from the *Chlamydomonas reinhardtii* perspective with a focus on distinctive features. *Curr. Genet.* 57, 151–168. 10.1007/s00294-011-0339-1 21533645

[B61] Tolleter D. Ghysels B. Alric J. Petroutsos D. Tolstygina I. Krawietz D. (2011). Control of hydrogen photoproduction by the proton gradient generated by cyclic electron flow in *Chlamydomonas reinhardtii*[W]. *Plant Cell* 23 2619–2630. 10.1105/tpc.111.086876 21764992 PMC3226202

[B62] Torzillo G. Scoma A. Faraloni C. Ena A. Johanningmeier U. (2009). Increased hydrogen photoproduction by means of a sulfur-deprived *Chlamydomonas reinhardtii* D1 protein mutant. *Int. J. Hydrogen Energy* 34 4529–4536. 10.1016/j.ijhydene.2008.07.093

[B63] Treves H. Küken A. Arrivault S. Ishihara H. Hoppe I. Erban A. (2021). Carbon flux through photosynthesis and central carbon metabolism show distinct patterns between algae, C3 and C4 plants. *Nat. Plants* 8 78–91. 10.1038/s41477-021-01042-5 34949804 PMC8786664

[B64] Volgusheva A. Styring S. Mamedov F. (2013). Increased photosystem II stability promotes H2 production in sulfur-deprived *Chlamydomonas reinhardtii*. *Proc. Natl. Acad. Sci. U.S.A.* 110 7223–7228. 10.1073/pnas.1220645110 23589846 PMC3645517

[B65] Weber A. Menzlaff E. Arbinger B. Gutensohn M. Eckerskorn C. Fluegge U.-I. (1995). The 2-oxoglutarate/malate translocator of chloroplast envelope membranes: molecular cloning of a transporter containing a 12-helix motif and expression of the functional protein in yeast cells. *Biochemistry* 34, 2621–2627. 10.1021/bi00008a028 7873543

[B66] Wenderoth I. Scheibe R. von Schaewen A. (1997). Identification of the cysteine residues involved in redox modification of plant plastidic glucose-6-phosphate dehydrogenase. *J. Biol. Chem.* 272 26985–26990. 10.1074/jbc.272.43.26985 9341136

[B67] Wiessner W. (1965). Quantum requirement for acetate assimilation and its significance for quantum measurements in photophosphorylation. *Nature* 205 56–57. 10.1038/205056a0 14283135

[B68] Xue X. Gauthier D. A. Turpin D. H. Weger H. G. (1996). Interactions between photosynthesis and respiration in the green alga *Chlamydomonas reinhardtii* (characterization of light-enhanced dark respiration). *Plant Physiol.* 112 1005–1014. 10.1104/pp.112.3.1005 12226429 PMC158027

[B69] Yamamoto H. Shikanai T. (2019). PGR5-dependent cyclic electron flow protects photosystem I under fluctuating light at donor and acceptor sides. *Plant Physiol.* 179 588–600. 10.1104/pp.18.01343 30464024 PMC6426425

