# Supplementary material for: Interactions Between Carbon Metabolism and Photosynthetic Electron Transport in a Chlamydomonas reinhardtii Mutant Without CO2 Fixation by RuBisCO
Source: Front Plant Sci. 2022 Apr 28;13:876439. doi: 10.3389/fpls.2022.876439 (PMC9096841; doi:10.3389/fpls.2022.876439)
Supplement: Supplementary Table 1 — Twenty-three chosen metabolites and their metabolic pathways. [file Table_1.DOCX]

| **Metabolites** | **Full name / Other names** | **Formula** | **Metabolic pathways (KEGG)** |
| --- | --- | --- | --- |
| **RuBP** | Ribulose-1,5-bisphosphate | C_5_H_12_O_11_P_2_ | Carbon fixation in photosynthetic organisms, Glyoxylate and dicarboxylate metabolism, Biosynthesis of secondary metabolites, Carbon metabolism |
| **S7P** | Sedoheptulose-7-phosphate | C_7_H_15_O_10_P | Carbon fixation in photosynthetic organisms, Pentose phosphate pathway, Lipopolysaccharide biosynthesis, Biosynthesis of secondary metabolites, Carbon metabolism, Biosynthesis of amino acids, Biosynthesis of nucleotide sugars |
| **R5P** | Ribose-5-phosphate | C_5_H_11_O_8_P | Carbon fixation in photosynthetic organisms, Pentose phosphate pathway, Purine metabolism, Phosphonate and phosphinate metabolism, Vitamin B6 metabolism, Biosynthesis of secondary metabolites, Carbon metabolism, Biosynthesis of amino acids |
| **Ru5P+Xu5P** | Ribulose 5-phosphate + Xylulose 5-phosphate | C_5_H_11_O_8_P x 2 | Carbon fixation in photosynthetic organisms, Pentose phosphate pathway, Pentose and glucuronate interconversions, Ascorbate and aldarate metabolism, Biosynthesis of secondary metabolites, Carbon metabolism, Biosynthesis of amino acids |
| **DHAP** | Dihydroxyacetone-phosphate | C_3_H_7_O_6_P | Glycolysis / gluconeogenesis, , Pentose and glucuronate interconversions, Fructose and mannose metabolism, Galactose metabolism, Glycerolipid metabolism, Inositol phosphate metabolism, Carbon fixation in photosynthetic organisms, Nicotinate and nicotinamide metabolism, Biosynthesis of secondary metabolites, Carbon metabolism, Biosynthesis of amino acids |
| **G6P** | Glucose 6-phosphate | C_6_H_13_O_9_P | Glycolysis / gluconeogenesis, Pentose phosphate pathway, Galactose metabolism, Amino sugar and nucleotide sugar metabolism, Biosynthesis of secondary metabolites, Biosynthesis of phenylpropanoids, Biosynthesis of alkaloids, Biosynthesis of nucleotide sugars, Carbon metabolism, AMPK signaling pathway |
| **F6P** | Fructose 6-phosphate | C_6_H_13_O_9_P | Starch and sucrose metabolism, Galactose metabolism, Carbon fixation in photosynthetic organisms, Biosynthesis of secondary metabolites, Carbon metabolism, AMPK signaling pathway |
| **G1P** | Glucose 1-phosphate | C_6_H_13_O_9_P | Glycolysis / gluconeogenesis, , Pentose and glucuronate interconversions, Galactose metabolism, Starch and sucrose metabolism, Amino sugar and nucleotide sugar metabolism, Glycerolipid metabolism, Biosynthesis of secondary metabolites, Biosynthesis of nucleotide sugars |
| **ADPG** | Adenosine-5'-Diphosphoglucose | C_16_H_23_N_5_Na_2_O_15_P_2_ | Starch and sucrose metabolism, Amino sugar and nucleotide sugar metabolism, Biosynthesis of secondary metabolites, Biosynthesis of nucleotide sugars |
| **NAD** | Nicotinamide adenine dinucleotide | C_21_H_27_N_7_O_14_P_2_ | Oxidative phosphorylation, Thiamine metabolism, Nicotinate and nicotinamide metabolism, AMPK signaling pathway |
| **NADP** | Nicotinamide adenine dinucleotide phosphate | C_21_H_29_N_7_O_17_P_3_ | Photosynthesis, Glutathione metabolism, Nicotinate and nicotinamide metabolism, |
| **ADP** | Adenosine Diphosphate | C_10_H_15_N_5_O_10_P2 | Oxidative phosphorylation, Photosynthesis, Purine metabolism, Zeatin biosynthesis, Biosynthesis of secondary metabolites, AMPK signaling pathway |
| **AMP** | Adenosine 5'-monophosphate | C_10_H_14_N_5_O_7_P | Purine metabolism, Zeatin biosynthesis, Biosynthesis of secondary metabolites, Biosynthesis of alkaloids, cAMP signaling pathway |
| **Glycerate** | D-Glycerate, Glyceric acid | C_3_H_6_O_4_ | Pentose phosphate pathway, Glycine, serine and threonine metabolism, Glycerolipid metabolism, Glyoxylate and dicarboxylate metabolism, Biosynthesis of secondary metabolites, Carbon metabolism |
| **Aconitate** | cis-Aconitate | C_6_H_6_O_6_ | Citrate cycle (TCA cycle), Glyoxylate and dicarboxylate metabolism, Biosynthesis of plant secondary metabolites, Biosynthesis of phenylpropanoids, Biosynthesis of terpenoids and steroids, Biosynthesis of alkaloids, Biosynthesis of secondary metabolites, 2-Oxocarboxylic acid metabolism |
| **Citrate** | Citric acid | C_6_H_8_O_7_ | Citrate cycle (TCA cycle), Alanine, aspartate and glutamate metabolism, Glyoxylate and dicarboxylate metabolism, Biosynthesis of plant secondary metabolites, Biosynthesis of phenylpropanoids, Biosynthesis of terpenoids and steroids, Biosynthesis of alkaloids, 2-Oxocarboxylic acid metabolism, Biosynthesis of amino acids |
| **Malate** | L-Malic acid | C_4_H_6_O_5_ | Citrate cycle (TCA cycle), Pyruvate metabolism, Glyoxylate and dicarboxylate metabolism, Carbon fixation in photosynthetic organisms, Biosynthesis of plant secondary metabolites, Biosynthesis of phenylpropanoids, Biosynthesis of terpenoids and steroids, Biosynthesis of alkaloids, Carbon metabolism |
| **Succinate** | Succinic acid | C_4_H_6_O_4_ | Citrate cycle (TCA cycle), Oxidative phosphorylation, Alanine, aspartate and glutamate metabolism, Glyoxylate and dicarboxylate metabolism, Lysine degradation, Tyrosine metabolism, Phenylalanine metabolism, Pyruvate metabolism, Nicotinate and nicotinamide metabolism, Sulfur metabolism, Biosynthesis of plant secondary metabolites, Biosynthesis of phenylpropanoids, Biosynthesis of terpenoids and steroids, Biosynthesis of alkaloids, Carbon metabolism, cAMP signaling pathway |
| **Isocitrate** | Isocitric acid | C_6_H_8_O_7_ | Citrate cycle (TCA cycle), Glyoxylate and dicarboxylate metabolism, Biosynthesis of plant secondary metabolites, Biosynthesis of phenylpropanoids, Biosynthesis of terpenoids and steroids, Biosynthesis of alkaloids, Carbon metabolism**,** 2-Oxocarboxylic acid metabolism, Biosynthesis of amino acids |
| **Oxoglutaric acid** | 2-Ketoglutaric acid | C_5_H_6_O_5_ | Citrate cycle (TCA cycle), Pentose and glucuronate interconversions, Ascorbate and aldarate metabolism, Arginine biosynthesis, Alanine, aspartate and glutamate metabolism, Lysine biosynthesis, Histidine metabolism, Glyoxylate and dicarboxylate metabolism, D-Amino acid metabolism, Biosynthesis of plant secondary metabolites, Biosynthesis of phenylpropanoids, Biosynthesis of terpenoids and steroids, Biosynthesis of alkaloids, Carbon metabolism**,** 2-Oxocarboxylic acid metabolism, Biosynthesis of amino acids |
| **Glutamate** | L-Glutamic acid | C_5_H_9_NO_4_ | Arginine biosynthesis, Alanine, aspartate and glutamate metabolism, Arginine and proline metabolism,  Histidine metabolism, D-Amino acid metabolism, Glutathione metabolism, Glyoxylate and dicarboxylate metabolism, Porphyrin and chlorophyll metabolism, Nitrogen metabolism, Biosynthesis of plant secondary metabolites, Biosynthesis of alkaloids, Carbon metabolism**,** 2-Oxocarboxylic acid metabolism |
| **Aspartate** | L-Aspartic acid | C_4_H_7_NO_4_ | Arginine biosynthesis, Alanine, aspartate and glutamate metabolism, Glycine, serine and threonine metabolism, Cysteine and methionine metabolism, Lysine biosynthesis, Histidine metabolism, D-Amino acid metabolism, Carbon fixation in photosynthetic organisms, Nicotinate and nicotinamide metabolism, Pantothenate and CoA biosynthesis, Biosynthesis of plant secondary metabolites, Biosynthesis of alkaloids, Carbon metabolism**,** 2-Oxocarboxylic acid metabolism**,** Biosynthesis of amino acids**,** ABC transporters |
| **UDPG** | UDP-glucose | C_15_H_24_N_2_O_17_P_2_ | Pentose and glucuronate interconversions, Galactose metabolism, Ascorbate and aldarate metabolism, Pyrimidine metabolism, Sartch and sucrose metabolism, Amino sugar and nucleotide sugar metabolism, Glycerolipid metabolism, Biosynthesis of secondary metabolites, Biosynthesis of nucleotide sugars |
