# Supplementary material for: Interactions Between Carbon Metabolism and Photosynthetic Electron Transport in a Chlamydomonas reinhardtii Mutant Without CO2 Fixation by RuBisCO
Source: Front Plant Sci. 2022 Apr 28;13:876439. doi: 10.3389/fpls.2022.876439 (PMC9096841; doi:10.3389/fpls.2022.876439)
Supplement: Supplementary Table 2 — Statistic tests explanations. [file Table_2.DOCX]

| **Statistical test** | **Test explanation** |
| --- | --- |
| Kruskal Wallis | Non-parametric alternative to ANOVA test, which extends the two-samples Wilcoxon test in the situation where there are more than two groups. |
| Dunn | Non-parametric pairwise multiple comparison test for quantitative data that identifies and classifies different types into different groups based on a variable. The test is based on the Bonferonni inequality. It is performed after a Kruskall-Wallis test. |
| Wilcoxon | Wilcoxon-Mann-Whitney test is a nonparametric statistical test (alternative to t test) that tests the hypothesis that the medians of each of two groups of data are close. Pairwise Wilcoxon test is the alternative of pairwise t test. |
| Anova | The one-way analysis of variance is an extension of independent two-samples t-test for comparing means in a situation where there are more than two groups. A two-way ANOVA is used to estimate how the mean of a quantitative variable changes according to the levels of two categorical variables. |
