# Supplementary material for: Interactions Between Carbon Metabolism and Photosynthetic Electron Transport in a Chlamydomonas reinhardtii Mutant Without CO2 Fixation by RuBisCO
Source: Front Plant Sci. 2022 Apr 28;13:876439. doi: 10.3389/fpls.2022.876439 (PMC9096841; doi:10.3389/fpls.2022.876439)
Supplement: Supplementary Table 3 — Metabolite data. [file Table_3.DOCX]

|  | **Metabolite** | **Replicate** | **Medium with acetate** *(Concentration nmol/10^7 cell)* | | | | | **Medium without acetate** *(Concentration nmol/10^7 cell)* | | | | |  |
| --- | --- | --- | --- | --- | --- | --- | --- | --- | --- | --- | --- | --- | --- |
|  |  |  | *dark* | *light* | | | *dark* | *dark* | *light* | | | *dark* | Light |
|  |  |  | ***0*** | ***10 sec*** | ***60*** | ***300*** | ***360*** | ***0,00*** | ***10 sec*** | ***60*** | ***300*** | ***360*** | ***Time (sec)*** |
| Calvin cycle / OPPP | RuBP | R1_1 | 0,16 | 0,17 | 0,37 | 0,97 | 1,12 | 0,35 | 1,16 | 2,00 | 4,47 | 4,53 |  |
|  |  | R1_2 | 0,13 | 0,19 | 0,34 | 1,20 | 1,35 | 0,66 | 0,68 | 2,87 | 5,46 | 4,45 |  |
|  |  | R2_1 | 0,77 | 0,92 | 1,60 | 3,41 | 2,89 | 0,70 | 2,00 | 2,62 | 5,66 | 6,40 |  |
|  |  | R2_2 | 0,73 | 1,08 | 1,68 | 2,44 |  | 0,87 | 1,98 | 3,50 | 7,85 |  |  |
|  | S7P | R1_1 | 0,06 | 0,08 | 0,06 | 0,07 | 0,08 | 0,04 | 0,03 | 0,00 | 0,01 | 0,03 |  |
|  |  | R1_2 | 0,05 | 0,07 | 0,06 | 0,07 | 0,08 | 0,04 | 0,04 | 0,02 | 0,02 | 0,03 |  |
|  |  | R2_1 | 0,07 | 0,07 | 0,06 | 0,09 | 0,07 | 0,06 | 0,03 | 0,00 | 0,01 | 0,06 |  |
|  |  | R2_2 | 0,06 | 0,08 | 0,06 | 0,05 |  | 0,04 | 0,02 | 0,01 | 0,01 |  |  |
|  | R5P | R1_1 | 0,08 | 0,08 | 0,07 | 0,08 | 0,07 | 0,03 | 0,03 | 0,02 | 0,03 | 0,04 |  |
|  |  | R1_2 | 0,05 | 0,08 | 0,10 | 0,08 | 0,09 | 0,02 | 0,04 | 0,03 | 0,03 | 0,03 |  |
|  |  | R2_1 | 0,09 | 0,07 | 0,08 | 0,05 | 0,08 | 0,03 | 0,05 | 0,01 | 0,01 | 0,03 |  |
|  |  | R2_2 | 0,05 | 0,08 | 0,08 | 0,04 |  | 0,02 | 0,04 | 0,02 | 0,02 |  |  |
|  | Ru5P+Xu5P | R1_1 | 0,04 | 0,07 | 0,06 | 0,11 | 0,06 | 0,02 | 0,07 | 0,02 | 0,04 | 0,07 |  |
|  |  | R1_2 | 0,07 | 0,07 | 0,05 | 0,08 | 0,08 | 0,02 | 0,05 | 0,04 | 0,03 | 0,04 |  |
|  |  | R2_1 | 0,09 | 0,09 | 0,09 | 0,05 | 0,11 | 0,02 | 0,08 | 0,01 | 0,02 | 0,06 |  |
|  |  | R2_2 | 0,03 | 0,08 | 0,08 | 0,04 |  | 0,02 | 0,06 | 0,01 | 0,03 |  |  |
| Gluconeogenesis / glycolysis | DHAP | R1_1 | 0,21 | 0,16 | 0,28 | 0,19 | 0,22 | 0,10 | 0,26 | 0,12 | 0,10 | 0,24 |  |
|  |  | R1_2 | 0,14 | 0,23 | 0,29 | 0,27 | 0,30 | 0,06 | 0,26 | 0,23 | 0,14 | 0,09 |  |
|  |  | R2_1 | 0,13 | 0,13 | 0,13 | 0,13 | 0,14 | 0,08 | 0,32 | 0,09 | 0,11 | 0,11 |  |
|  |  | R2_2 | 0,08 | 0,15 | 0,13 | 0,11 |  | 0,07 | 0,30 | 0,10 | 0,18 |  |  |
|  | G6P | R1_1 | 0,56 | 0,79 | 0,82 | 0,74 | 0,81 | 0,70 | 0,79 | 0,47 | 0,41 | 0,76 |  |
|  |  | R1_2 | 0,61 | 0,99 | 0,99 | 1,14 | 1,09 | 0,58 | 0,84 | 0,98 | 0,66 | 0,57 |  |
|  |  | R2_1 | 0,88 | 1,08 | 1,47 | 1,40 | 1,01 | 0,79 | 0,88 | 0,54 | 0,49 | 0,72 |  |
|  |  | R2_2 | 0,64 | 1,10 | 1,11 | 1,02 |  | 0,67 | 0,71 | 0,47 | 0,52 |  |  |
|  | F6P | R1_1 | 0,21 | 0,29 | 0,28 | 0,31 | 0,30 | 0,18 | 0,25 | 0,16 | 0,12 | 0,22 |  |
|  |  | R1_2 | 0,23 | 0,29 | 0,27 | 0,38 | 0,36 | 0,18 | 0,23 | 0,31 | 0,16 | 0,14 |  |
|  |  | R2_1 | 0,30 | 0,38 | 0,50 | 0,49 | 0,39 | 0,23 | 0,24 | 0,14 | 0,15 | 0,21 |  |
|  |  | R2_2 | 0,20 | 0,41 | 0,43 | 0,37 |  | 0,19 | 0,21 | 0,15 | 0,18 |  |  |
|  | G1P | R1_1 | 0,06 | 0,07 | 0,08 | 0,08 | 0,07 | 0,08 | 0,07 | 0,07 | 0,06 | 0,09 |  |
|  |  | R1_2 | 0,07 | 0,10 | 0,10 | 0,12 | 0,09 | 0,06 | 0,08 | 0,09 | 0,07 | 0,07 |  |
|  |  | R2_1 | 0,06 | 0,13 | 0,12 | 0,13 | 0,11 | 0,09 | 0,10 | 0,07 | 0,08 | 0,09 |  |
|  |  | R2_2 | 0,05 | 0,09 | 0,12 | 0,09 |  | 0,07 | 0,08 | 0,07 | 0,09 |  |  |
| Starch | ADPG | R1_1 | 0,02 | 0,03 | 0,01 | 0,01 | 0,02 | 0,04 | 0,05 | 0,01 | 0,01 | 0,03 |  |
|  |  | R1_2 | 0,01 | 0,01 | 0,01 | 0,02 | 0,02 | 0,03 | 0,03 | 0,02 | 0,03 | 0,04 |  |
|  |  | R2_1 | 0,04 | 0,06 | 0,05 | 0,09 | 0,07 | 0,03 | 0,03 | 0,01 | 0,01 | 0,08 |  |
|  |  | R2_2 | 0,04 | 0,07 | 0,06 | 0,04 |  | 0,02 | 0,02 | 0,02 | 0,02 |  |  |
| Energy precursors | NAD | R1_1 | 1,98 | 2,31 | 1,71 | 2,13 | 1,92 | 2,69 | 2,06 | 1,50 | 1,49 | 1,79 |  |
|  |  | R1_2 | 1,59 | 1,88 | 1,77 | 1,87 | 2,03 | 2,40 | 2,91 | 2,31 | 2,38 | 1,14 |  |
|  |  | R2_1 | 2,09 | 2,37 | 2,47 | 3,10 | 2,16 | 1,72 | 1,57 | 1,41 | 1,25 | 1,91 |  |
|  |  | R2_2 | 2,01 | 3,08 | 2,56 | 1,63 |  | 1,20 | 1,35 | 1,57 | 1,95 |  |  |
|  | NADP | R1_1 | 1,36 | 1,41 | 1,02 | 1,27 | 1,00 | 0,41 | 0,36 | 0,26 | 0,29 | 0,34 |  |
|  |  | R1_2 | 0,80 | 0,97 | 0,96 | 0,88 | 1,07 | 0,41 | 0,45 | 0,37 | 0,49 | 0,24 |  |
|  |  | R2_1 | 0,87 | 0,92 | 0,84 | 1,29 | 1,00 | 0,33 | 0,31 | 0,23 | 0,25 | 0,40 |  |
|  |  | R2_2 | 0,68 | 1,14 | 1,00 | 0,59 |  | 0,27 | 0,30 | 0,35 | 0,40 |  |  |
|  | ADP | R1_1 | 3,26 | 2,48 | 2,47 | 2,57 | 2,15 | 2,22 | 1,06 | 0,96 | 0,80 | 2,25 |  |
|  |  | R1_2 | 1,60 | 1,95 | 2,25 | 2,21 | 2,53 | 2,00 | 1,53 | 1,55 | 1,51 | 1,36 |  |
|  |  | R2_1 | 3,18 | 2,26 | 2,44 | 3,12 | 2,78 | 1,70 | 0,84 | 0,64 | 0,71 | 2,40 |  |
|  |  | R2_2 | 2,29 | 2,76 | 2,52 | 1,55 |  | 1,46 | 0,67 | 1,04 | 1,13 |  |  |
| Sugar acids | glycerate | R1_1 | 1,63 | 1,94 | 1,92 | 1,78 | 2,04 | 0,80 | 0,71 | 0,71 | 1,03 | 0,75 |  |
|  |  | R1_2 | 1,63 | 1,95 | 1,98 | 2,22 | 2,24 | 0,61 | 0,72 | 0,90 | 0,66 | 0,49 |  |
|  |  | R2_1 | 1,46 | 1,52 | 1,34 | 1,28 | 1,31 | 0,58 | 0,56 | 0,45 | 0,48 | 0,50 |  |
|  |  | R2_2 | 1,04 | 1,34 | 1,32 | 1,08 |  | 0,51 | 0,52 | 0,41 | 0,58 |  |  |
| TCA / glyoxylate cycle | aconitate | R1_1 | 0,44 | 0,48 | 0,42 | 0,43 | 0,45 | 0,02 | 0,03 | 0,02 | 0,03 | 0,03 |  |
|  |  | R1_2 | 0,42 | 0,51 | 0,49 | 0,49 | 0,55 | 0,02 | 0,02 | 0,02 | 0,02 | 0,02 |  |
|  |  | R2_1 | 0,34 | 0,38 | 0,39 | 0,35 | 0,31 | 0,02 | 0,02 | 0,02 | 0,02 | 0,02 |  |
|  |  | R2_2 | 0,28 | 0,41 | 0,38 | 0,26 |  | 0,02 | 0,01 | 0,01 | 0,02 |  |  |
|  | citrate | R1_1 | 59,43 | 62,27 | 50,11 | 61,00 | 55,01 | 3,71 | 4,34 | 3,78 | 3,08 | 3,85 |  |
|  |  | R1_2 | 43,92 | 53,86 | 50,63 | 51,93 | 56,91 | 5,57 | 4,71 | 4,09 | 4,41 | 4,10 |  |
|  |  | R2_1 | 17,12 | 16,86 | 17,27 | 23,53 | 18,13 | 3,89 | 3,72 | 2,76 | 3,38 | 5,53 |  |
|  |  | R2_2 | 14,83 | 22,99 | 18,07 | 12,04 |  | 3,28 | 3,03 | 3,50 | 5,25 |  |  |
|  | isocitrate | R1_1 | 0,07 | 0,06 | 0,04 | 0,09 | 0,06 | 0,07 | 0,07 | 0,04 | 0,06 | 0,04 |  |
|  |  | R1_2 | 0,04 | 0,04 | 0,03 | 0,03 | 0,03 | 0,06 | 0,06 | 0,04 | 0,08 | 0,04 |  |
|  |  | R2_1 | 0,12 | 0,17 | 0,16 | 0,17 | 0,16 | 0,04 | 0,04 | 0,03 | 0,04 | 0,07 |  |
|  |  | R2_2 | 0,12 | 0,15 | 0,12 | 0,11 |  | 0,02 | 0,04 | 0,04 | 0,08 |  |  |
|  | succinate | R1_1 | 98,19 | 116,41 | 110,50 | 100,16 | 112,32 | 1,59 | 1,16 | 1,26 | 2,44 | 1,87 |  |
|  |  | R1_2 | 100,30 | 133,26 | 121,34 | 135,56 | 151,53 | 1,39 | 1,25 | 1,89 | 1,97 | 0,89 |  |
|  |  | R2_1 | 21,57 | 21,34 | 23,98 | 21,05 | 19,01 | 1,35 | 0,75 | 0,97 | 0,72 | 1,13 |  |
|  |  | R2_2 | 16,17 | 22,55 | 20,68 | 15,84 |  | 0,82 | 1,07 | 0,75 | 1,11 |  |  |
|  | malate | R1_1 | 388,88 | 458,72 | 439,42 | 397,38 | 448,94 | 2,33 | 2,48 | 2,32 | 2,45 | 3,11 |  |
|  |  | R1_2 | 387,06 | 530,81 | 503,07 | 522,24 | 593,19 | 1,65 | 2,31 | 4,14 | 2,14 | 1,65 |  |
|  |  | R2_1 | 62,00 | 64,56 | 70,57 | 61,32 | 57,44 | 1,63 | 1,69 | 1,28 | 1,39 | 2,15 |  |
|  |  | R2_2 | 45,31 | 66,54 | 62,62 | 45,21 |  | 1,37 | 1,46 | 1,30 | 1,65 |  |  |
|  | 2og | R1_1 | 18,71 | 21,32 | 20,11 | 19,74 | 20,91 | 1,99 | 1,77 | 1,25 | 1,40 | 1,81 |  |
|  |  | R1_2 | 17,40 | 24,32 | 23,93 | 24,45 | 28,33 | 1,41 | 2,08 | 2,43 | 1,78 | 0,82 |  |
|  |  | R2_1 | 6,74 | 6,25 | 6,50 | 6,49 | 5,75 | 1,08 | 1,14 | 0,95 | 0,93 | 1,11 |  |
|  |  | R2_2 | 4,50 | 6,38 | 6,18 | 4,83 |  | 0,97 | 1,02 | 0,87 | 1,28 |  |  |
| Amino acids | glutamate | R1_1 | 21,82 | 25,66 | 25,02 | 22,95 | 26,42 | 4,75 | 5,26 | 4,44 | 6,59 | 4,79 |  |
|  |  | R1_2 | 21,53 | 30,33 | 28,21 | 30,79 | 33,68 | 4,06 | 4,78 | 5,98 | 4,52 | 2,47 |  |
|  |  | R2_1 | 15,58 | 16,02 | 17,85 | 16,76 | 14,32 | 2,91 | 3,23 | 2,86 | 3,01 | 3,06 |  |
|  |  | R2_2 | 10,74 | 15,72 | 14,91 | 12,51 |  | 2,45 | 2,79 | 2,65 | 3,71 |  |  |
|  | aspartate | R1_1 | 44,09 | 51,62 | 50,71 | 44,51 | 53,63 | 5,31 | 5,08 | 9,04 | 22,10 | 5,37 |  |
|  |  | R1_2 | 44,66 | 60,77 | 53,63 | 61,68 | 69,50 | 4,56 | 4,87 | 6,91 | 4,17 | 4,76 |  |
|  |  | R2_1 | 19,43 | 15,52 | 17,66 | 17,42 | 15,24 | 5,64 | 5,16 | 4,23 | 4,34 | 5,52 |  |
|  |  | R2_2 | 12,39 | 16,12 | 14,63 | 11,41 |  | 4,35 | 4,43 | 4,34 | 5,28 |  |  |
|  | UDPG | R1_1 | 0,05 | 0,08 | 0,13 | 0,15 | 0,13 | 0,41 | 0,43 | 0,44 | 0,35 | 0,31 |  |
|  |  | R1_2 | 0,05 | 0,11 | 0,17 | 0,24 | 0,16 | 0,33 | 0,39 | 0,73 | 0,44 | 0,27 |  |
|  |  | R2_1 | 0,32 | 0,37 | 0,59 | 0,64 | 0,36 | 0,53 | 0,57 | 0,55 | 0,40 | 0,36 |  |
|  |  | R2_2 | 0,25 | 0,38 | 0,54 | 0,44 |  | 0,43 | 0,47 | 0,51 | 0,50 |  |  |
| Signalling | AMP | R1_1 | 5,91 | 6,36 | 6,58 | 6,57 | 7,66 | 0,78 | 0,51 | 0,41 | 0,31 | 1,57 |  |
|  |  | R1_2 | 5,58 | 8,25 | 7,63 | 7,60 | 9,46 | 0,60 | 0,55 | 0,68 | 0,41 | 0,63 |  |
|  |  | R2_1 | 2,29 | 2,31 | 2,57 | 2,32 | 2,58 | 0,47 | 0,30 | 0,24 | 0,19 | 1,08 |  |
|  |  | R2_2 | 1,72 | 2,22 | 2,20 | 1,82 |  | 0,45 | 0,24 | 0,27 | 0,32 |  |  |
